# Polarity and anti-distortive polarons in WO_3_ through epitaxial shear strain

**DOI:** 10.1038/s41467-026-75049-x

**Published:** 2026-07-13

**Authors:** Ewout van der Veer, Martin F. Sarott, Jack T. Eckstein, Stijn Feringa, Dennis van der Veen, Johanna van Gent González, Majid Ahmadi, Horatio R. J. Cox, Ellen M. Kiens, Gertjan Koster, Bart J. Kooi, Michael A. Carpenter, Ekhard K. H. Salje, Beatriz Noheda

**Affiliations:** 1 Zernike Institute for Advanced Materials, University of Groningen, Groningen, Netherlands; 2 Faculty of Physics, University of Duisburg-Essen, Duisburg, Germany; 3 Research Center Future Energy Materials and Systems (RC FEMS), Research Alliance Ruhr, Bochum, Germany; 4 Groningen Cognitive Systems and Materials Center (CogniGron), University of Groningen, Groningen, The Netherlands; 5 Department of Earth Sciences, University of Cambridge, Cambridge, UK; 6 Center for Nanophase Materials Sciences, Oak Ridge National Laboratory, Oak Ridge, TN USA; 7 MESA+ Institute for Nanotechnology, University of Twente, Enschede, The Netherlands

**Keywords:** Ferroelectrics and multiferroics, Electronic devices, Electronic properties and materials

## Abstract

Bestowing complementary metal-oxide semiconductor-compatible binary oxides with additional functionalities is a powerful strategy toward the realization of oxide electronics. Ideal candidates are thin films which display a strong sensitivity to strain, chemical doping or nanoscale confinement. Among these, crystalline tungsten trioxide WO_3_ exhibits exceptional structural flexibility, enabling a wide range of functionalities. Here we reveal the emergence of a polar phase in epitaxial WO_3_ thin films. We accomplish this by imposing epitaxial shear strain, which stabilizes a low-symmetry triclinic structure that persists up to large film thicknesses and elevated temperatures. At the atomic scale, a change in the oxygen octahedral tilt pattern facilitates this symmetry lowering into a polar phase, which manifests as a periodic in-plane polarized stripe domain configuration with needle-like bifurcations at the microscale. The stripe domain walls further exhibit a strongly enhanced electrical conductivity in conjunction with a pronounced reduction of a distortive structural mode, providing experimental evidence for anti-distortive polaronic transport recently predicted in WO_3_.

## Abstract (web summary)

WO_3_ single crystals are known to exhibit a large number of structural polymorphs. Here, the authors study epitaxial WO_3_ thin films and provide evidence for a polar phase driven by epitaxial shear strain as well as evidence of anti-distortive polarons.

## Introduction

Transition metal oxides exhibit a wide range of physical properties, making them attractive candidates for new electronic materials. Real-world applications of this class of materials have, however, long been limited by their difficult integration with existing complementary metal-oxide semiconductor (CMOS) fabrication processes. Recent efforts have demonstrated additional useful functionalities in simpler binary transition metal oxides that are innately CMOS-compatible via chemical doping, interface engineering, nanoscale confinement, or strain engineering strategies. Nanoscale ferroelectricity in hafnia^1^ and the metal-to-insulator transition in vanadium dioxide^2^ are the most prominent examples. Another similar binary oxide is tungsten trioxide (WO_3_), which is conventionally used for its electrochromic^3,4^ and gas sensing^5^ properties, as well as increasingly in resistive random access memory (ReRAM) devices^6,7^, though predominantly in amorphous or polycrystalline form.

In single crystal form, WO_3_ exhibits a large number of structural polymorphs, as a result of its relatively simple structure consisting of corner-sharing WO_6_ octahedra. The structure of WO_3_ can be thought of as a perovskite structure (i.e., ABO_3_) with a vacant A-site, commonly known as the ReO_3_ structure. Within this general framework, there exist various different phases, distinguished by their respective patterns of octahedral tilts and distortions. These phases and the phase transitions between them, have been extensively reviewed in literature^8–14^, with each of them being ferroelastic, leading to complex twinning patterns. Of the known phases, only the lowest-temperature *ϵ* phase (*T* < 240 K, space group *Pc*) has been reported to be (weakly) polar^15,16^, though other accounts have suggested that the ground state phase has a centrosymmetric structure with space group *P*2_1_/*n*^13,14,17,18^. Furthermore, the boundaries between ferroelastic domains in WO_3_ have shown intriguing functionalities not present in bulk, including locally enhanced conductivity^19^, superconductivity^20–23^ and a recently discovered electrical polarization, induced by local strain gradients at the twin walls through an effect termed *flexopiezoelectricity*^17,24–26^.

Despite having an extraordinary structural flexibility and remarkable electronic properties, WO_3_ has, thus far, mostly been studied in bulk, single-crystal samples, rather than in crystalline thin films, which are more relevant for applications in electronic devices. Moreover, using epitaxial strain engineering to stabilize metastable phases, manipulate the domain configuration, or act on the domain-wall density in WO_3_ tends to be hindered by the sparsity of lattice-matching substrates. Note that the room-temperature stable phase in bulk WO_3_ displays a *P*2_1_/*n* monoclinic symmetry with *a* = 7.306 Å, *b* = 7.522 Å, *c* = 7.678 Å, and *β* = 90.88°, resulting in pseudo-cubic lattice parameters of *a*_*pc*_ = 3.653 Å, *b*_*pc*_ = 3.761 Å and *c*_*pc*_ = 3.839 Å. Consequently, for thin films of WO_3_ grown on most standard oxide substrates, even on those with small lattice parameters, such as sapphire^27,28^, SrTiO_3_^28–32^, LSAT^30,33^, or LaAlO_3_^30,32,34^, the lattice mismatch is too large for epitaxial growth to be achieved and the WO_3_ films grow unstrained and contain a large amount of structural defects. Exceptions are (110)-oriented YAlO_3_ (YAO) substrates. Coherently strained films have been successfully grown on these substrates^24,25,32,33^. However, in this case, no deviations from the bulk stable monoclinic *P*2_1_/*n* phase have been observed.

In this work, we show that it is possible to stabilize a polar and piezoelectric phase in epitaxial WO_3_ thin films at room temperature. This polar phase is the result of a symmetry lowering from monoclinic to triclinic, driven by epitaxial shear strain imposed when growing WO_3_ on (001)-oriented YAO substrates. Using a combination of scanning transmission electron microscopy (STEM), angle-resolved piezoresponse force microscopy (PFM), and large-scale X-ray diffraction (XRD) reciprocal space mapping, we determine the structure of this polar phase from the atomic scale to the macroscale. We find that the WO_3_ films consist of four equivalent triclinic domain variants that give rise to a stripe-like piezoelectric domain structure with an in-plane orientation of the polarization. At the atomic level, the triclinic polar phase is characterized by a unique octahedral tilt pattern that gets progressively suppressed in the vicinity of the domain walls. Furthermore, conductive atomic force microscopy (cAFM) and scanning electron microscopy (SEM) reveal an enhanced electrical conductivity at these domain walls despite the absence of a polar discontinuity at these domain walls. Together with the marked reduction of the distortive structural mode at the domain walls unveiled by STEM, our electrical characterization provides experimental evidence for the existence of anti-distortive polarons, recently predicted by Hassani et al.^35^, which have been associated with superconductivity^36^.

## Results and Discussion

### Polar domain structure

**Fig. 1:**
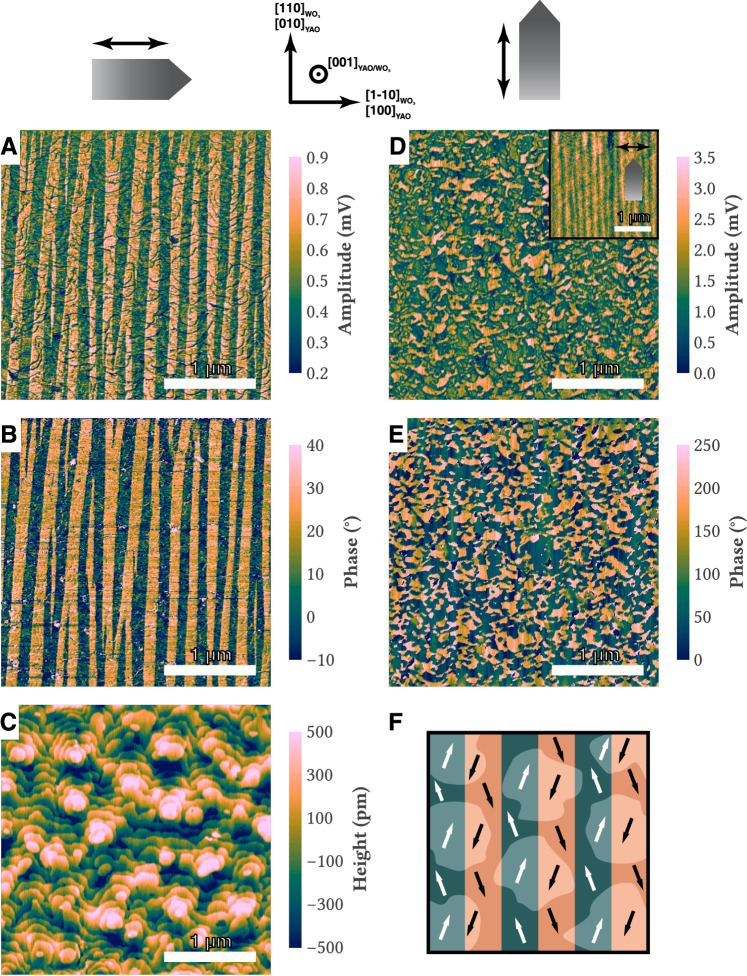
Angle-resolved lateral piezoresponse force microscopy (PFM) of a WO_3_ film on a (001)-oriented YAlO_3_ substrate. Sample rotations of (**A**, **B**) 0° and (**D**, **E**) 90° with respect to the reference axes displayed on the top, showing lateral PFM (**A**, **D**) amplitude and (**B**, **D**) phase. The inset in (**D**) shows a vertical PFM amplitude image with a stripe-like buckling contrast corresponding to the domain configuration seen in (**A**), obtained via a 90° rotation of the fast scanning axis. **C** Sample topography. **F** Schematic illustration of the polarization vector. The polarization is predominantly oriented along the stripe domains seen in (**A**, **B**) with a small component in the orthogonal direction (**D**, **E**).

We begin our investigation of the WO_3_ films, grown via pulsed laser deposition on (001)-oriented YAO (see Methods), by performing atomic force microscopy (AFM) and angle-dependent piezoresponse force microscopy (PFM), as shown in Fig. 1. For a film with a thickness of 157 nm, the AFM topography (Fig. 1C) shows an atomically flat surface with a wedding-cake-like morphology that is characteristic of two-dimensional layer-by-layer growth and in accordance with reflection high-energy electron diffraction measurements acquired in-situ during thin-film growth (see Supplementary Fig. S2). The lateral PFM phase and amplitude images, taken with the cantilever axis oriented along the [100]_O_ direction of the orthorhombic YAO substrate (see Fig. 1A–B), reveal clear domain contrast with long stripe-like domains and domain walls along the [010]_O_ direction of the YAO. Since lateral PFM is sensitive to a projected polarization perpendicular to the cantilever axis, such a domain contrast indicates the presence of an in-plane polarization that lies along the stripe-domains and alternates in sign between neighboring domains. The deviation from the expected 180° phase contrast may be explained by a slight misalignment of the laser above the probe tip, an effect which is especially pronounced in weakly piezoelectric samples^37^. Supplementary Fig. S3 shows an additional PFM scan with a 180° contrast between oppositely polarized domains.

To deepen our understanding of this domain configuration, we rotate the sample with respect to the cantilever axis and perform angle-dependent lateral PFM, as shown in Fig. 1D–E and Supplementary Figs. S4–S6. Upon rotating the sample by 90°, we detect additional mosaic-like domain features with an in-plane polarization component that lies perpendicular to that of the stripe domains along [100]_O_ of YAO. Note that when changing the fast scanning axis to scan across the stripe domains for the film rotated by 90°, the stripe domains reappear in the vertical channel as a buckling contrast (see inset of Fig. 1D), which is consistent with an in-plane polarization as previously reported in other materials^38,39^. Hence, as illustrated in Fig. 1F, our lateral PFM data reveal the presence of a periodic stripe domain pattern in the WO_3_ films with an in-plane polarization that predominately lies along [010]_YAO_ and exhibits a small component along [100]_YAO_, with irregular, meandering domain walls. No domain contrast is found in vertical PFM (see Supplementary Fig. S4), but a small enhancement of the out-of-plane piezoresponse is present at the domain walls. This local response is likely related to strain gradient-induced flexopiezoelectricity, as proposed by Yun et al.^25^ and observed in WO_3_ films on (110)-oriented YAO. Note that despite the piezoelectric response of the WO_3_ films, we do not observe an optical second harmonic generation (SHG) signal due to a macroscopic cancellation of the net polarization, which eliminates any SHG emission through destructive interference^40^.

Observing such a highly anisotropic domain pattern is rather surprising, given that the pseudo-cubic (001) cut of the YAO substrate does not have an in-plane anisotropy along the pseudocubic unit cell axes. Instead, the domain anisotropy likely originates entirely from the non-orthogonal base angle (91. 5°) of the substrate and the resulting epitaxial shear strain imposed on the WO_3_ film. Indeed, no comparable anisotropic domain pattern is observed in WO_3_ films on (110)-oriented YAO^24,25^, where the two in-plane lattice constants are different (*a*_*pc*_ = 3.68 Å vs. *b*_*pc*_ = 3.71 Å), but the base angle is 90° and, thus, no shear strain is imposed. The strong dependence of the polar domain structure on the YAO base angle suggests that a shear strain-induced distortion of the WO_3_ unit cell is the primary driving force for the anisotropic domain formation (see Supplementary Note 1).

The domain pattern further exhibits needle-like bifurcations, a feature commonly found in uniaxial polar and ferroelectric materials^41–43^. For instance, the stripe-like domain pattern found here is reminiscent of that found in thin samples of BaTiO_3_ prepared by focused ion beam milling^41^. In this system, the domain walls are vertical and along the in-plane polarization direction, so the domain walls maintain zero net charge. New domains nucleate at sharp bifurcation points within domains of opposite polarity to minimize the surface area of the resulting charged domain wall, thereby minimizing the energy cost associated with the resulting uncompensated bound charge. Note that tip-induced manipulation of the domain configuration using a DC electric field or tip pressure has been unsuccessful due to the relatively high conductivity of the WO_3_ film and the absence of a suitable bottom electrode.

### Macroscopic structural characterization

**Fig. 2:**
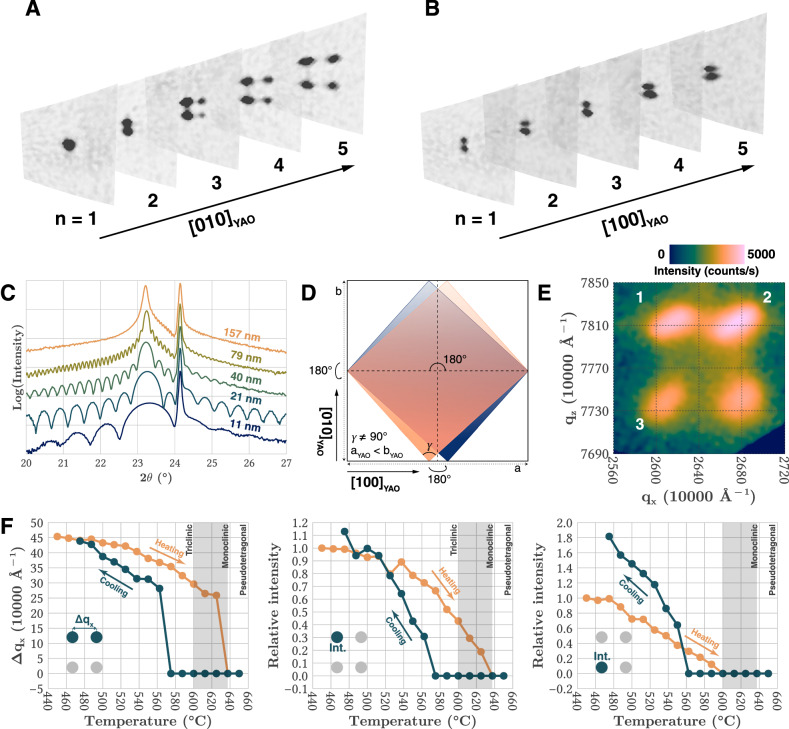
Structural characterization of WO_3_ films on (001)-oriented YAO. Projections of reciprocal lattice planes of increasing order of a 157 nm WO_3_ film along the **A** [010]_YAO_ and the [100]_YAO_ directions. Each image represents the a) (2+n $\bar{1}$+n 4) or **B** ($\bar{1}$+n 2-n 4) WO_3_ reflections, using pseudo-orthorhombic indices. The vertical and horizontal directions in each image are **A** [001]_YAO_ and [100]_YAO_; and **B** [001]_YAO_ and [010]_YAO_, respectively. The images are presented in grayscale, with white and black representing minimum and maximum intensity, respectively. **C** Symmetric *θ*-2*θ* XRD line scans of the (002) reflection of WO_3_ films with varying thicknesses. **D** Diagram showing the relationship between two structural WO_3_ domains and the YAO substrate. The color gradient indicates an out-of-plane tilt of the film unit cell around the [100]_YAO_ axis. The other two domains are related to the ones shown here by a 180° rotation about the [001] axis perpendicular to the page. **E** Reciprocal space map of the pseudo-cubic 103 reflections of the WO_3_ film at 450 °C showing the four triclinic domains. The spots labeled '1', '2', and '3' were used to define the order parameters in (**F**). **F** Evolution of the order parameters of the phase transition from the low-temperature triclinic phase into the high-temperature pseudo-orthorhombic phase.

In bulk crystals of WO_3_^13^, a monoclinic phase with space group *P*2_1_/*n* is stable at room temperature. This phase is centrosymmetric and non-piezoelectric. The formation of in-plane polarized domains with a distinctive piezoresponse (Fig. 1) is, therefore, rather unexpected. To shed light on the structural origins of this polar phase, we perform large scale reciprocal space mapping using a single-crystal X-ray diffractometer (see Methods) on a 157 nm thick WO_3_ film (see Fig. 2). Figure 2A shows reconstructed images of reciprocal space around the (2+n $\bar{1}$+n 4) and ($\bar{1}$+n 2-n 4) reflections belonging to lattice planes spanned by the [001] (vertical in the image) and [100] (horizontal in the image) crystallographic directions of the YAO unit cell at different offsets along the [010] direction, corresponding to the 1^st^ through the 5^th^ order of the same diffraction peak. The WO_3_ peak clearly splits into four equivalent peaks in a square arrangement with increasing distance along [010]_YAO_. Figure 2B shows the equivalent planes along the [100]_YAO_ direction (90° rotated around the [001] axis normal to the sample surface), where a two-fold vertical splitting is seen (identical to the vertical splitting along the [010]_YAO_ direction in the image labeled *n* = 2), but no change with increasing diffraction order. The lack of increasing peak splitting with increasing diffraction order along the [100]_YAO_ axis shows that the observed peak splitting cannot originate from symmetric tilting of a higher-symmetry unit cell (e.g., monoclinic) with respect to the substrate without deformation of that cell into one of a lower (i.e., triclinic) symmetry. A reconstruction of the full reciprocal lattice planes perpendicular to these two directions is shown in Supplementary Fig. S7. This pattern is inconsistent with the monoclinic *P*2_1_/*n* phase in bulk WO_3_ (see also Supplementary Fig. S8), where crystal twins would give rise to two-fold splitting along [001] or [100] (leading to four-fold splitting in a different arrangement).

The observed peak splitting (the peak positions and the evolution of the splitting with the amplitude of the q-vector) can only be explained if the symmetry of the crystal lattice is triclinic. We indexed the peaks using the CrysAlisPro software package into four equivalent triclinic domains, with the lattice parameters listed in Table 1 (the measured orientation matrices and twin laws are provided in Supplementary Note 2, equivalent simulated diffraction patterns in Supplementary Figs. S8 and S9, and the relative orientations of the domains with respect to each other in real space are shown in Supplementary Fig. S10).

**Table 1:** Crystallographic data for WO_3_ film on (001)-oriented YAlO_3_

Temperature	300 K
Lattice system	Triclinic
Wavelength	1.54056 Å
*ϕ* range	0° – 360°
2*θ* range collected	0.46° – 140.54°
**Index limits**	
*h*	− 8 to 8
*k*	− 8 to 8
*l*	1 to 5
**Lattice parameters**	
a	7.323 ± 0.006 Å
b	7.470 ± 0.002 Å
c	7.682 ± 0.002 Å
*α*	89.04 ± 0.03°
*β*	89.16 ± 0.03°
*γ*	88.74 ± 0.05°
V	419.8 ± 0.4 Å^3^

A triclinic (space group $P\bar{1}$) phase is known to exist in bulk WO_3_ between 230–290 K, but the lattice parameters and unit cell volume of this reported phase are different from those measured here^8–10^. Also, the existence of a center of inversion in the reported $P\bar{1}$ phase precludes the presence of a piezoresponse and polar domains, as we observe it in Fig. 1, which implies a *P1* space group in our films.

Symmetric *θ*-2*θ* scans (Fig. 2C) show extended Laue oscillations over the full range of thicknesses, indicating that the films are of high quality and crystallinity. Films with thicknesses ranging from 11 to 157 nm show no detectable differences in the lattice parameter, or other signatures of strain relaxation, indicating that the same phase observed in the thickest film is present already in the thinner films (see also Supplementary Fig. S11).

Figure 2D illustrates the epitaxial relation between the YAO substrate (black square) and two of the triclinic domains of the WO_3_ film, showing how the non-90°*γ* angle results in peak splitting along the [100]_YAO_ axis. The peaks are further split along the [001]_YAO_ axis due to the deviation from 90° of the other two unit cell angles. The resulting additional two domains are shown in Supplementary Fig. S12). Hence, each diffraction spot in Fig. 2A corresponds to one of the four structural domains (see also Supplementary Figs. S8, S9 and S10). The domains are related to each other by 180° rotations about the three principal axes of the orthorhombic unit cell of the substrate. The [100]_YAO_ and [001]_YAO_ axes are collinear with the ${[1\bar{1}0]}_{{{{{\rm{WO}}}}}_{3}}$ and ${[001]}_{{{{{\rm{WO}}}}}_{3}}$ axes, respectively.

We further characterize the temperature stability of the *P1* phase by temperature-dependent X-ray reciprocal space mapping (RSM) around the (226) peak of the WO_3_ film (equivalent to the (103) peak of a pseudo-cubic perovskite). In the room-temperature triclinic phase, the RSM contains four diffraction spots in a square arrangement, again belonging to the four structural domains (see Fig. 2E and Supplementary Fig. S13). Upon heating, we observe a phase transition around 600 °C, above which two diffraction spots, belonging to the high-temperature phase, remain. To describe this phase transition in more detail, we define three different order parameters: (1) The separation between the top two diffraction spots, (2) the normalized intensity of the top-left diffraction spot and (3) the normalized intensity of the bottom-left diffraction spot. The latter two intensities are measured relative to the intensity of the top-right diffraction spot to correct for any overall decrease of intensity due to misalignment or sample degradation. The evolution of these order parameters is shown in Fig. 2F. We find that the triclinic phase is maintained up to a temperature of 600 °C, after which the film undergoes a broad, two-step phase transition that is completed at 650 °C. The bottom two spots disappear in the first step around 600 °C on heating (order parameter 3), resulting in either a domain configuration with two triclinic domains or a monoclinic intermediate phase. The top spots decrease in intensity and merge at around 640 °C (order parameters 1 and 2) while two new diffraction spots appear belonging to a high-temperature phase with orthorhombic or tetragonal symmetry. Upon cooling, the triclinic spots reappear in the reverse order starting at 580 °C. The intensity of the top spot returns to the same level as before the transition, whereas that of the bottom spot increases by a factor of almost two, indicating that the population of the different structural domains in the sample changes upon cycling through the transition (see Supplementary Fig. S13). Both on heating and on cooling, there exists a broad temperature range in which the diffraction spots of the low-temperature triclinic phase and the high-temperature phase are both present, indicating phase coexistence (see Supplementary Fig. S13 and the Supplementary Video). In addition, the three order parameters in Fig. 2F each exhibit temperature hysteresis. Both of these observations suggest that the phase transition between the triclinic and the high-temperature phase is of a discontinuous, first-order nature.

The series of phase transitions found in our films differs markedly from that reported in bulk WO_3_^13^, for which the monoclinic *P*2_1_/*n* phase is stable between 290 K and 338 K and the only known triclinic $P\bar{1}$ phase exists between 230–290 K. The difference in the phase transition temperature seen here demonstrates that the epitaxial shear strain plays a fundamental role in the stability of the triclinic *P1* phase in the WO_3_ films.

### Nanostructural characterization

**Fig. 3:**
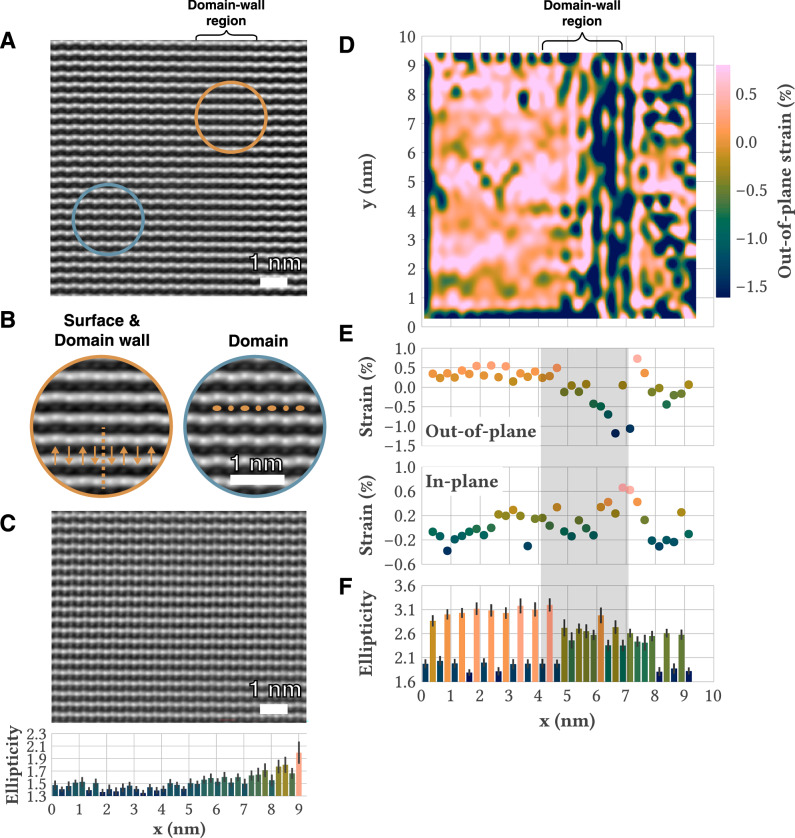
Atomic-resolution structural characterization of the WO_3_ film on (001)-oriented YAO. **A** iDPC-STEM images of the film near a domain wall. **B** Blow-ups of the image in (**A**) showing the change of structure in the surface and domain-wall region and in the interior of a domain. Arrows in the left panel show the direction of the zigzag modulation of W atomic column positions. A half-unit-cell phase shift of this modulation occurs at the dashed line. **C** iDPC-STEM image along the in-plane zone axis perpendicular to that in (**A**), showing no modulation of the oxygen column ellipticity (shown at the bottom of the image). The colors of the bars are correlated with the magnitude of the ellipticity. All iDPC-STEM images are presented in grayscale, black and white representing minimum and maximum projected electrostatic potential, respectively. **D** Map of the out-of-plane (i.e., vertical in the image) strain in the image in (**A**). **E** Vertically-averaged profiles of the out-of-plane and in-plane strain in the image in (**A**) showing out-of-plane contraction and in-plane expansion of the unit cell at the domain wall. The symbol colors are correlated to the strain magnitude. **F** Vertically-averaged ellipticity of atomic columns of oxygen in the V_A_Olayers showing a modulation of the ellipticity in the interior of the domains, which disappears in the domain-wall region. The error bars in (**C**, **F**) represent the standard error of the averaged values of the ellipticity.

Having established symmetry lowering into a triclinic phase as the structural origin of the emergence of polar domains in Fig. 1, we next move to scanning transmission electron microscopy (STEM) measurements to gain insights into the atomic-scale mechanism for strain accommodation. Figure 3A shows an integrated differential phase contrast (iDPC)-STEM image of the film along the [110] zone axis near a domain wall, showing sublayers of WO_2_ and V_A_O, where V_A_ are the vacancies in the A-sites related to the perovskite structure. The WO_2_ sublayers exhibit a zigzag-like modulation of W atomic positions that originates from the antipolar M3 distortion present in all reported low-symmetry phases of WO_3_ (see also Supplementary Fig. S14)^13^. A progressive suppression of this zigzag modulation is visible in a vertical region (indicated in Fig. 3A as “domain-wall region") of about eight pseudo-cubic unit cells in width. This region is associated with a half-unit-cell phase shift of the modulation, *i.e*. the region to the left of the domain wall is related to that on the right by a 180° rotation about the ${[110]}_{{{{{\rm{WO}}}}}_{3}}$ (zone axis) direction or the ${[1\bar{1}0]}_{{{{{\rm{WO}}}}}_{3}}$ direction (horizontal in the image). Such a 180° rotation is exactly the relation between two structural twins found by X-ray diffraction (Fig. 2D), suggesting that this region of significantly reduced W-column modulation corresponds to a structural domain wall and, in particular, the domain wall between two polar stripe domains in Fig. 1B. Careful tracing of the atomic columns allows us to pinpoint the exact position where the distortion changes direction and shows that the actual twin wall is in fact a single unit cell thick (left panel in Fig. 3B). A similar suppression of the zigzag modulation is also found in the first three to four unit cells near the surface of the film (Supplementary Fig. S15).

To further investigate the structural changes in the film near the domain wall, we perform real-space image analysis on the STEM image (see Supplementary Note 3) in Fig. 3A and extract the local lattice parameters (i.e., strain) as a function of the position in the image, as well as the ellipticities of oxygen atomic columns in the V_A_Osublayers. Figure 3D shows a map of the out-of-plane strain across the domain wall. The out-of-plane lattice parameter decreases in the vicinity of the domain wall while it is constant in the interior of the domain. Integrating the strain map in the vertical direction yields the strain profile across the domain wall in Fig. 3E (top panel). A profile of the in-plane strain made using the same procedure is shown in the bottom panel. The contraction of the out-of-plane lattice parameter near the domain wall (gray shaded region in Fig. 3E–F) is associated with an expansion of the in-plane lattice parameter.

Furthermore, in the interior of the domains, we find that every second atomic column of oxygen in the V_A_Osublayers is broadened in the horizontal direction (see also Supplementary Fig. S20). That is, they exhibit an alternating broad/narrow pattern of ellipticity represented by broadened ellipses in Fig. 3B (right panel). The magnitude of the ellipticity of each oxygen column in the V_A_Osublayer is measured by real-space image analysis and integrated over each vertical line to yield the bottom profile shown in Fig. 3F. This alternating pattern of ellipticity—a direct consequence of the oxygen octahedral tilt pattern—is consistent with a triclinic *P1* phase but inconsistent with the expected octahedral tilting patterns of both the monoclinic (*P*2_1_/*n*) and bulk triclinic ($P\bar{1}$) phases (see Supplementary Fig. S14)^44,45^. Moreover, as discernible in Fig. 3F, in the vicinity of the domain wall (gray band), this ellipticity alternation is markedly suppressed, along with the reduction of the zigzag motion of the W atomic columns. Fig. 3C shows an iDPC-STEM image along the [1$\bar{1}$0] zone axis (*i.e*. orthogonal to the STEM image in (a)) and corresponding oxygen column ellipticities. In this zone, no comparable modulation of the ellipticities is present, demonstrating a clear structural anisotropy. Advanced (synchrotron) x-ray diffraction techniques as well as DFT calculations may further elucidate the exact atomic origin of the polarization in this structure, but these are beyond the scope of the current work. Our STEM data, thus, show an anisotropic polar structure with distinct higher-symmetry domain walls characterized by strongly reduced structural distortions.

### Domain width scaling with film thickness

**Fig. 4:**
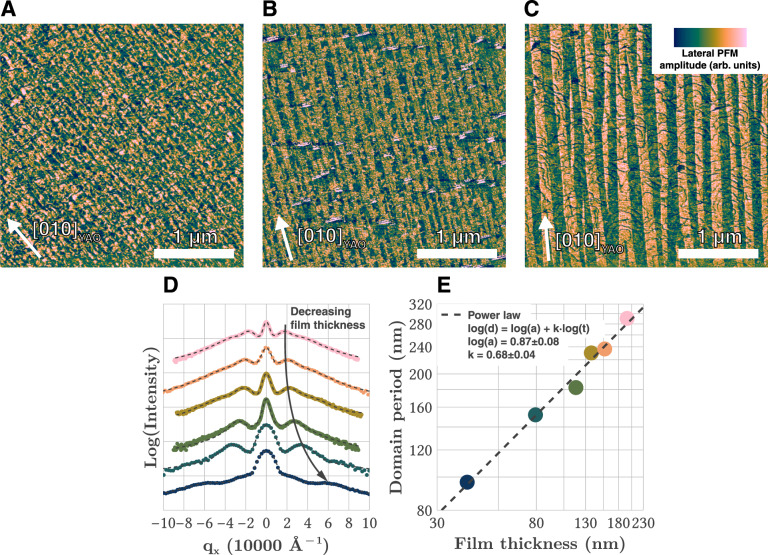
Domain width dependence on film thickness. Lateral PFM amplitude images for a (**A**) 40 nm, **B** 79 nm and **C** 157 nm thick film. The arrows represent the predominant in-plane polarization axis. **D** *ω* rocking curves around the (004) peak of the WO_3_ films, showing pronounced thickness-dependent domain satellite peaks. q_x_ corresponds to the (1$\bar{1}$0) axis of the WO_3_ film. The dashed line represents the fit to the data. **E** Log-log plot of the domain periodicity as a function of film thickness, extracted from the domain satellite peak positions in (**D**). The dashed line is a fit to the data. The colors in (**D**–**E**) show the correspondence between the data points in (**E**) with the profiles in (**D**).

The dependence of domain size on film thickness can provide additional valuable insight into the driving force for the formation of a ferroelectric or ferroelastic domain structure and can demonstrate the ability for a material to be used in nanoscale electronics. To investigate the domain size scaling in our WO_3_ films, we consider films with thicknesses ranging between 40 and 196 nm and quantify the domain size in these films using PFM and X-ray *ω* rocking curves around the (004) peak of the WO_3_ film. For large film thicknesses (*t*_film_ ≫ *d*_DW_), the domain size in ferroelectrics and ferroelastics usually follows a power law with a scaling exponent of *γ* = 0.5, in analogy with Kittel’s law for ferromagnets^46,47^. Figure 4A–C shows lateral PFM images of films with different thicknesses, where the stripe domain width clearly increases with growing film thickness. This is further confirmed by the X-ray *ω* rocking curve analysis, shown in Fig. 4D. Here, we observe the presence of pronounced satellite peaks in the [100]_YAO_ direction with the peak position depending on the film thickness. These peaks do not appear in the orthogonal [010]_YAO_ direction and, therefore, must originate from the periodic stripe domains. The domain period extracted from the spacing of the satellite peaks is, indeed, consistent with that observed in PFM (Fig. 4E). A power law fit to this data yields a scaling exponent of 0.68 ± 0.04, similar to the value found for WO_3_ films on (110)-oriented YAO^24^.

Similar deviations from classical Kittel scaling (*γ* = 0.5) have been seen in cases where domain walls are broadened due to magnetoelectric coupling^48^ and in the thin-film (i.e., *t*_film_ ≈ *d*_DW_) regime due to ferroelastic effects and interactions between the substrate-film interface and the film surface^49–51^. In our case, we do indeed also observe a widening of the domain walls (see Fig. 3A) that likely results from coupling between the structural and polar order parameters, giving rise to a strain gradient at the domain walls. The triclinic phase persists up to the thickest investigated film with a thickness of 92 nm (see Supplementary Fig. S16).

### Domain-wall conductivity and evidence for anti-distortive polarons

**Fig. 5:**
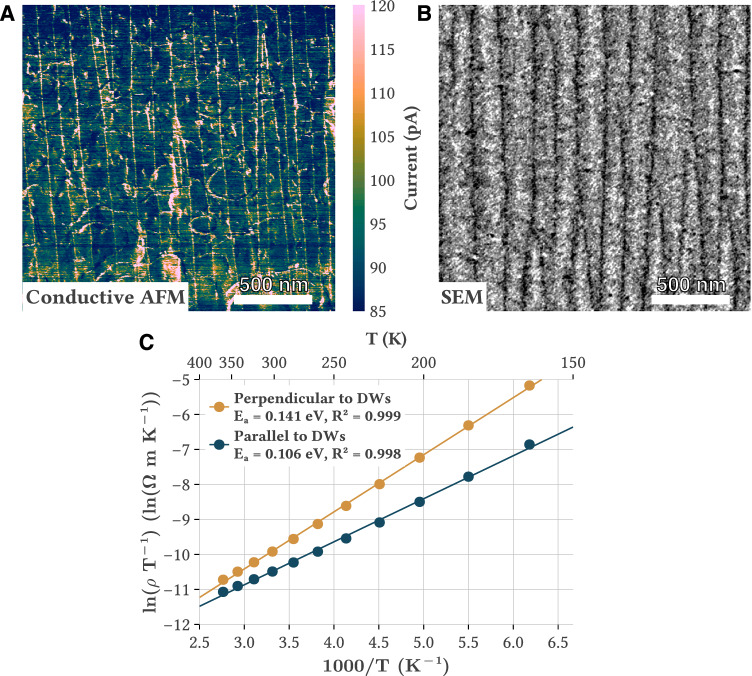
Micro- and macroscopic transport characteristics of the triclinic stripe domain walls in WO_3_. **A** cAFM image showing domain-wall contrast with an enhanced domain-wall conductivity. **B** SEM image showing a reduced secondary electron yield at the location of the stripe domain walls. The image is presented in grayscale, black and white representing minimum and maximum secondary electron yield, respectively. **C** Temperature-dependent conductivity parallel and perpendicular to the stripe domain walls measured using Hall bars (bottom panel of Supplementary Fig. S17). The solid lines represent the fit according to a polaron transport model^59^.

Domain walls in ferroic materials are characterized by a distinctly different symmetry than the interior of a domain, which can give rise to the emergence of additional functional properties^52,53^. Moreover, domain walls in polar and ferroelectric materials frequently represent polar discontinuities that can repel or attract internal charge carriers or defects, and thus, exhibit locally modified conductance^54,55^. Even in ferroelastic materials, the conductivity of twin domain walls can be locally changed because of the accumulation of charged defects, such as oxygen vacancies^19^. To probe the local conducting properties of the stripe domain walls in our triclinic and polar WO_3_ films, we use conductive atomic force microscopy (cAFM) and scanning electron microscopy (SEM), as shown in Fig. 5A–B. The cAFM image in Fig. 5A reveals an increased conductivity of the domain walls compared to the bulk of the domains, which appears further enhanced in the vicinity of bifurcations. We also observe a minor increase of the detected current at topographical step edges, likely due to the locally increased tip-sample contact area. The SEM image, shown in Fig. 5B, provides a complementary view of the local conductivity differences. Here, we identify a clear decrease of the secondary electron yield at the domain walls. Such a reduction is in good agreement with the expectation for a highly conductive region in which the primary electron beam is preferentially conducted away and, thus, the escape probability of secondary electrons is lowered^56,57^. For in-plane ferroelectrics, the formation of charged head-to-head or tail-to-tail domain walls can lead to conducting domain walls and domain-wall contrast in SEM as previously reported^58^. In our WO_3_ films, however, the domain walls are neutral without a polar discontinuity, which points to a different conduction mechanism at play. Recent theoretical work by Hassani et al. ^35^ proposes the formation of anti-distortive polarons in WO_3_, which are associated with a reduction of the structural distortions. Their work further suggests that this reduction of structural distortions is accompanied by a lowering of the conduction band minimum due to improved hybridization between O2p and W5d orbitals. The reduced structural distortion seen at the domain walls in our films (see Fig. 3) can be viewed as a static manifestation of the same effect that is present dynamically in polaronic charge transport. The atomic structure of a mobile polaron can, however, not be probed directly by any currently available structural characterization method.

To gain insight into the conduction mechanism in our samples, we consider temperature-dependent electrical transport measurements parallel and perpendicular to the stripe domains using Hall bars, as shown in Fig. 5C and Supplementary Fig. S17. The temperature-dependent conductivity in Fig. 5C can be well fitted with a polaron transport model^59^ (see Methods) for both orientations, whereas neither simple band gap activation nor variable range hopping can reproduce the data. We further observe a distinctly reduced activation energy for transport parallel to the domain walls (*E*_*a*,∥_ = 0.106 ± 0.002 eV) in comparison to the direction perpendicular to the domain walls (*E*_*a*,⊥_ = 0.141 ± 0.001 eV) as well as a reduced charge carrier mobility although a polaronic conduction mechanism is at play in both cases. This anisotropy can be attributed to the perpendicular path forcing charge carriers to hop from the conductive domain wall into the bulk of the domains, a process which constitutes a higher energy barrier than moving along the domain boundary network itself. Consistent with Hassani et al.^35^, we suggest that the increased conductivity at domain walls in our film is due to a combination of band gap lowering associated with the reduction of octahedral tilting^35^ and facilitation of anti-distortive polaron formation in the domain-wall region. Thus, given the clear indication for macroscopic polaronic transport in combination with enhanced domain wall conductivity and the undoing of the octahedral tilts at the domain walls, our work constitutes experimental evidence for the formation of anti-distortive polarons. In conclusion, we have demonstrated the stabilization of a polar phase in thin films of WO_3_ grown on (001)-oriented YAO. These films exhibit a stripe-like domain configuration with a near uniaxial in-plane polarization and needle-like bifurcations. Structurally, this polar WO_3_ phase originates from a symmetry lowering into a triclinic system that is characterized by a unique pattern of oxygen octahedral tilts and gives rise to four structural domains. Remarkably, we discover that the neutral WO_3_ stripe-domain walls exhibit enhanced conductivity, while simultaneously featuring a reduction of the distortive atomic modes inherent to the triclinic phase. This combination, hence, provides experimental evidence for the existence of anti-distortive polarons, as predicted theoretically. These results constitute an important step towards the realization of domain wall-based nanoelectronics.

## Methods

### Thin film growth

WO_3_ thin films were grown on the (001) face of as-received single-crystal substrates of YAO (Crystec GmbH) by pulsed laser deposition using a 248 nm KrF excimer laser. Films were grown from a ceramic WO_3_ target at a temperature of 680°C, an oxygen pressure of 0.1 mbar, a laser fluence of 0.5 J, cm^−2^ (spot size 2.1 mm^2^), laser repetition rate of 2 Hz and a target-substrate distance of 50 mm. After growth, the films were kept at 680°C for up to 30 min, then cooled to room temperature at a rate of 10 ${}^{\circ }{{{\rm{C}}}}\,{\min }^{-1}$ in growth pressure. A total of between 200 and 20000 pulses were deposited, yielding final film thicknesses of 5–392 nm (see Supplementary Fig. S18). The films were shown by x-ray photoelectron spectroscopy to consist of stoichiometric WO_3_ (Supplementary Fig. S19).

### X-ray diffraction

Large-scale reciprocal space mapping was performed on a Bruker D8 Venture diffractometer equipped with a I*μ* S Cu microfocus X-ray source and a Photon II detector with sample placed at a 20° angle of incidence to the X-ray beam. Data are collected as two 360°*ϕ* scans about an axis normal to the sample surface with a static detector placed at 2*θ* = 36° and 105°, similar to the method described by Sønsteby et al.^60^ 2*θ*-*ω* line scans, reciprocal space maps and *ω* rocking curves were collected using a Panalytical X’Pert MRD thin-film diffractometer using a Cu source with a 2xGe(220) hybrid monochromator and a PIXcel^3D^ area detector in scanning line mode (2*θ*-*ω* line scans) or receiving slit mode (*ω* rocking curves). Fits were made to the rocking curves by least squares fitting of pseudo-Voigt functions. Additional 2*θ*-*ω* scans and reciprocal space maps were measured with a Bruker D8 Discover diffractometer equipped with a rotating anode X-ray source (Cu-K*α* radiation), a Dectris Eiger2 R 500K two-dimensional area detector, a Ge(220) monochromator, and a 1 mm beam collimator. For 2*θ*-*ω*, the detector was used in 0D mode with a region of interest of 9 × 55 pixels and a fixed detector distance of 295 mm. For reciprocal space maps, the detector was operated in 1D mode and kept stationary, while performing an *ω* rocking curve.

### Electron microscopy

Scanning electron microscopy images were collected on a FEI Helios G4 CX scanning electron microscope with an accellerating voltage of 2 kV and a beam current of 1.4 nA. Cross-section (${[110]}_{{{{{\rm{WO}}}}}_{3}}$ zone) TEM lamellae were prepared by focussed ion beam milling (FEI Helios G4 CX) and Ga^+^ ion polishing at 5 kV and 2 kV to a thickness of approximately 100 nm. The TEM samples were plasma cleaned in 25%/75% O_2_/Ar atmosphere for 1 min just before imaging. A double-aberration-corrected ThermoFisher Themis Z scanning transmission electron microscope was operated at 300 kV with a beam current of 50 pA and a beam semiangle of 24 mrad. High-angle annular dark field (HAADF) and integrated differential phase contrast^61^ (iDPC) images were collected simultaneously at collection angles of 27–164 mrad and 7–25 mrad, respectively. A four-segment annular dark field detector was used to collect iDPC images. The effect of drift was removed by drift-corrected frame integration (an additional iDPC-STEM image, not using drift correction, is shown in Supplementary Fig. S20). Samples were imaged in the ${[110]}_{{{{{\rm{WO}}}}}_{3}}$ (cross-section) zone.

### PFM and cAFM

Scanning probe microscopy measurements were performed on an Asylum Research Cypher ES AFM using Bruker SCM-PIC-V2 PtIr-coated conductive AFM probes. PFM measurements were done in air and on-resonance with a tip-sample force of approximately 15 nN. Lateral and vertical PFM images were collected with the long axis of the cantilever along the [100], [010] and [110] in-plane directions of the substrate. Additional measurements were performed using stiffer PtIr-coated (Bruker SCM-PIT-V2) and CoCr-coated (Bruker MESP-RC-V2) probes to exclude tip-specific artifacts which can arise when using soft probes (such as SCM-PIC-V2). cAFM measurements were performed on the same instrument with SCM-PIC-V2 probes. A small amount of silver paint was applied to part of the surface of the film, to which a positive electrical bias of 200–800 mV was applied. The current flowing from the sample into the grounded probe was measured while scanning.

### Hall bar fabrication and electrical measurements

Hall bar devices were fabricated on the WO_3_ films using a two-step electron-beam lithography (EBL) process, which defined channels with dimensions of 150 × 30 *μ*m. The procedure involved the e-beam evaporation and liftoff of Ti (5 nm)/Au (40 nm) electrodes, followed by Ar ion-beam etching to isolate the active channel. Temperature- and field-dependent transport properties were measured in a Quantum Design Physical Property Measurement System (PPMS) within a low-pressure ( < 4 mbar) N_2_ environment from 100–400 K. Electrical contacts were made using ultrasonic Al wire bonding. At each temperature, a Keithley 6221 Current Source and Agilent 3458A Voltmeter were used to perform four-probe measurements while sweeping the magnetic field from  − 9 T to +9 T. Intrinsic electronic transport parameters were extracted using a two-stage linear regression analysis of the raw voltage data. The process involved performing current sweeps at discrete magnetic fields, followed by a sweep of the field itself at each temperature. To further improve accuracy and eliminate geometric errors, measurements were systematically performed across two sets of opposing voltage terminals. This dual-sweep and multi-terminal methodology enables the systematic removal of extrinsic voltage offsets, including thermal and geometric artifacts, as detailed by Lindemuth^62^. More details on the fitting procedure and the full analysis code are available on GitHub^63^.

### Data processing

The CrysAlisPro software package, commonly used for single-crystal x-ray diffraction indexing and refinement, was used to process single-crystal XRD data, to fit the lattice parameters of the four domains and to make simulated precession images from the data. The measurement geometry used does not allow for the accurate determination of diffraction peak intensities, so only peak positions are used. The STEM data were processed using ThermoFisher Velox software and in-house software^64^. SEM data were processed using the Fiji^65^ software package. The image was cropped and rotated, then denoised using Fourier filtering and Gaussian convolution. Contrast in the image was enhanced using the contrast limited adaptive histogram equalization (CLAHE) method^66^. PFM and cAFM data were processed using the Gwyddion^67^ software package. The images were leveled by aligning rows and subtracting a second order polynomial from each. Horizontal scan artifacts were removed. The images were cropped and rotated where necessary to orient the stripe domains vertically. All further processing and fitting of data was done using the Julia programming language^68^ using the GLM package^69^ for fitting. Data were plotted using the Makie package^70^ with scientific color maps designed by Crameri^71^.

## Supplementary information

Supplementary Information

Description of Additional Supplementary Files

Supplementary Video 1

Transparent Peer Review file

## Data Availability

The data supporting this study are available in DataverseNL at 10.34894/QDT8NH.
