# Supplementary material for: Polarity and anti-distortive polarons in WO3 through epitaxial shear strain
Source: Nat Commun. 2026 Jul 13;17:8593. doi: 10.1038/s41467-026-75049-x (PMC13486495; doi:10.1038/s41467-026-75049-x)
Supplement: Supplementary file 1 [file 41467_2026_75049_MOESM1_ESM.pdf]

Supplementary information for: Polarity and anti-distortive polarons in WO_3 through epitaxial shear strain

Ewout van der Veer‡ * [1,2,3], Martin F. Sarott‡ [1,4], Jack T. Eckstein [5,6], Stijn Feringa [1], Dennis van der Veen [1], Johanna van Gent González [1], Majid Ahmadi [1,4], Horatio R. J. Cox [1,4], Ellen M. Kiens [7], Gertjan Koster [7], Bart J. Kooi [1,4], Michael A. Carpenter [5], Ekhard K. H. Salje† [5], and Beatriz Noheda * [1,4]

[1]Zernike Institute for Advanced Materials, University of Groningen, Groningen, The Netherlands

[2]Faculty of Physics, University of Duisburg-Essen, Duisburg, Germany

[3]Research Center Future Energy Materials and Systems (RC FEMS), Research Alliance Ruhr, Bochum, Germany

[4]Groningen Cognitive Systems and Materials Center (CogniGron), University of Groningen, Groningen, The Netherlands

[5]Department of Earth Sciences, University of Cambridge, Cambridge, United Kingdom

[6]Center for Nanophase Materials Sciences, Oak Ridge National Laboratory, Oak Ridge, Tennessee, USA

[7]MESA+ Institute for Nanotechnology, University of Twente, Enschede, The Netherlands

* Corresponding authors. Email: ewout.vanderveer@uni-due.de (E.vdV.), b.noheda@rug.nl (B.N.)

‡ These authors contributed equally to this work.

Supplementary Note S1 Epitaxial strain calculation

To quantify the magnitude of the linear and shear strains applied to the WO_3 film by the (001) facet (our films) and the (110) facet (previous work by Yun *et al.*[1,2]) of $YAlO_3$, we use the procedure outlined in ref. [3]. We calculate the epitaxial strain with respect to two potential reference phases: 1) the known bulk monoclinic phase stable at room temperature and 2) a hypothetical cubic phase with the same unit cell volume. Tab. S1 lists the linear (ε_{11} and ε_{22}) and the shear (ε_{12}) strains applied to the film when grown on the (001) face of $YAlO_3$, showing that the magnitude of the shear strain is comparable to that of the linear strains. In contrast, when grown on the (110) face of $YAlO_3$ (Tab. S2), the shear strains are zero, while the linear strains are of similar magnitude to those on the (001) face. These results demonstrate that epitaxial shear strain plays a crucial role in stabilizing the polar triclinic phase.

Table S1: Strain tensor components for a WO_3 film grown on (001)-terminated $YAlO_3$ with respect to the bulk monoclinic and a hypothetical cubic reference phase.

**YAO(001)**	Monoclinic reference cell	Cubic reference cell
ε_{11}	0.01229	-0.01494
ε_{22}	-0.01878	-0.01530
ε_{12}	0.01355	0.01318

Table S2: Strain tensor components for a WO_3 film grown on (110)-terminated $YAlO_3$ with respect to the bulk monoclinic and a hypothetical cubic reference phase. The tensor components are reported separately for the A and B domains as defined by Yun *et al.*[2]

**YAO(110)**	Monoclinic A domain	Monoclinic B domain	Cubic reference cell
ε_{11}	-0.00490	0.01265	-0.01494
ε_{22}	-0.01878	-0.02629	-0.02248
ε_{12}	0	0	0

To determine the evolution of the strain tensor components with temperature, we calculated the lattice parameters of the $YAlO_3$ substrate and the monoclinic and cubic reference phases of WO_3 at temperatures between room temperature and 700 °C using coefficients of thermal expansion from refs. [4, 5]. The strain tensor components were then again calculated using the procedure from ref. [3], the results of which are shown in Fig. S1.

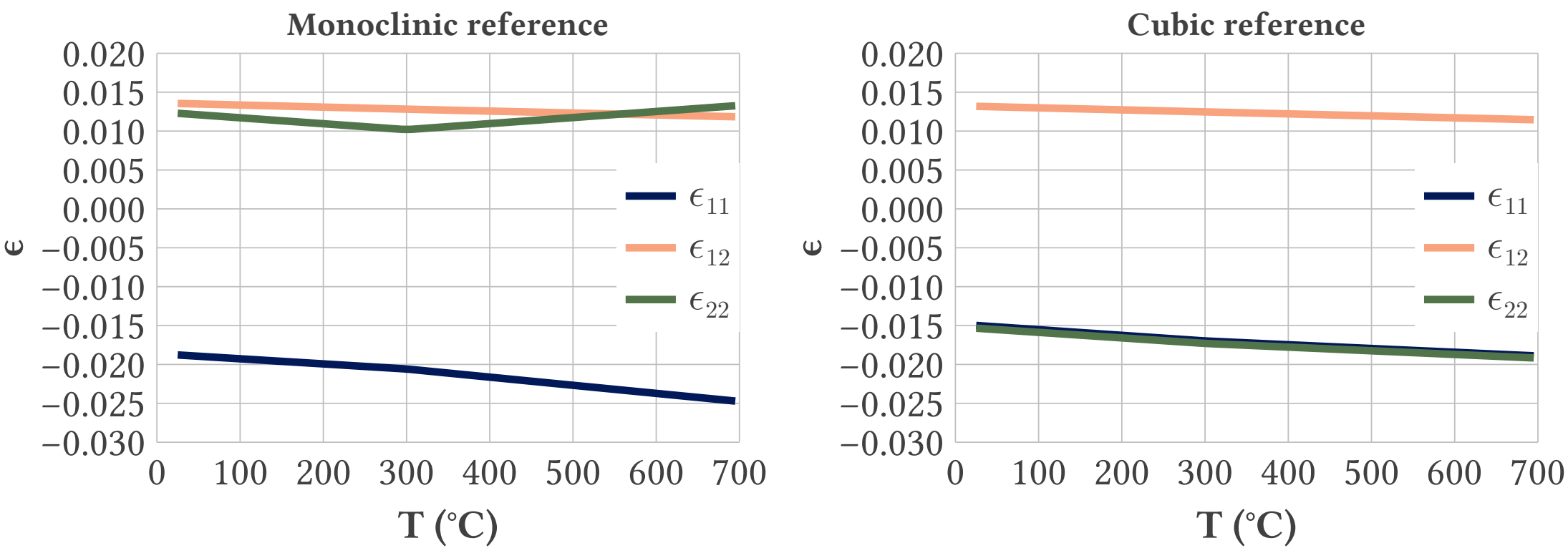

Figure S1: Evolution of strain tensor components with temperature. Calculated using the procedure described in Supplementary Note S1. Left panel: strain with respect to monoclinic reference structure. Right panel: strain with respect to hypothetical cubic reference structure.

Supplementary Note S2 Orientation matrices of structural domains

The collected single-crystal x-ray diffraction dataset was processed using the CrysAlisPro software package. After loading the data, the diffraction peak positions were found using the "Traditional peak hunting" method with single-frame background subtraction. The detected diffraction peaks were indexed into four equivalent domains. Strongly overlapping peaks were removed from the dataset, after which the lattice parameters were refined on the remaining data. The resulting (experimental) orientation matrices of the four domains are listed below:

$$\mathbf{UB_1} = \begin{pmatrix} 0.15868 & 0.13206 & -0.00115 \\ 0.13845 & -0.15854 & 0.00060 \\ 0.00250 & 0.00215 & -0.20065 \end{pmatrix} \quad \mathbf{UB_2} = \begin{pmatrix} -0.13153 & -0.15818 & 0.00115 \\ 0.16405 & -0.13262 & -0.00028 \\ -0.00210 & -0.00255 & 0.20058 \end{pmatrix}$$

$$\mathbf{UB_3} = \begin{pmatrix} -0.15871 & -0.13205 & -0.00116 \\ -0.13839 & 0.15844 & 0.00017 \\ 0.00382 & 0.00424 & -0.20055 \end{pmatrix} \quad \mathbf{UB_4} = \begin{pmatrix} 0.13153 & 0.15811 & 0.00109 \\ -0.16401 & 0.13233 & -0.00070 \\ -0.00420 & -0.00388 & 0.20055 \end{pmatrix}$$

The twin laws relating these four domains are then calculated to be:

$$\mathbf{T_{1\to 2}} = \mathbf{UB_2^{-1}} \cdot \mathbf{UB_1} = \begin{pmatrix} 0.01972 & -0.98153 & 0.00116 \\ -1.01956 & -0.0187 & -0.00097 \\ -0.00029 & 0.0002 & -1.00035 \end{pmatrix} \approx \begin{pmatrix} 0 & -1 & 0 \\ -1 & 0 & 0 \\ 0 & 0 & -1 \end{pmatrix}$$

$$\mathbf{T_{1\to 3}} = \mathbf{UB_3^{-1}} \cdot \mathbf{UB_1} = \begin{pmatrix} -0.99996 & 0.00039 & -0.00135 \\ 0.00045 & -1.00026 & 0.00154 \\ -0.0315 & -0.03186 & 1.00051 \end{pmatrix} \approx \begin{pmatrix} -1 & 0 & 0 \\ 0 & -1 & 0 \\ 0 & 0 & 1 \end{pmatrix}$$

$$\mathbf{T_{1\to 4}} = \mathbf{UB_4^{-1}} \cdot \mathbf{UB_1} = \begin{pmatrix} -0.02077 & 0.98148 & 0.00018 \\ 1.02067 & 0.01854 & -0.00053 \\ 0.03178 & 0.03163 & -1.00051 \end{pmatrix} \approx \begin{pmatrix} 0 & 1 & 0 \\ 1 & 0 & 0 \\ 0 & 0 & -1 \end{pmatrix}$$

These results show that the domains are related to each other by 180° rotations about the $[1\bar{1}0]$ ($\mathbf{T_{1\to 2}}$), [001] ($\mathbf{T_{1\to 3}}$) and [110] ($\mathbf{T_{1\to 4}}$) axes.

Supplementary Note S3 STEM image processing

The scanning transmission electron microscopy image presented in Fig. 3 was processed and analyzed using an in-house developed real-space analysis software package called STEMfit. The analysis procedure using STEMfit comprises the following steps:

1. Image filtering

   Image filtering is done by truncated singular value decomposition, in which the experimental image is decomposed according to $\mathbf{M} = \mathbf{U}\mathbf{\Sigma}\mathbf{V}^{\intercal}$, where $\mathbf{\Sigma}$ is a diagonal matrix of singular values. This matrix is truncated after a limited number of values (usually 30-70), after which the STEM image is reconstructed with the truncated matrix $\mathbf{\Sigma}'$. Any remaining high-frequency noise is removed by gaussian smoothing with a 2-3 pixel kernel.

2. Image thresholding

   The filtered image is subsequently segmented using Niblack[6] adaptive local thresholding. For each pixel, a threshold value is calculated using $t_{ij} = \mu_N + (b \cdot \sigma_N)$ where μ_N is the average pixel value of all pixels in a window N around the pixel at i, j, b is a thresholding parameter called *bias* and σ_N is the standard deviation of all pixels in window N.

3. Atomic column detection

   Pixels with a value $> t_{ij}$ form contiguous areas corresponding to the atomic columns in the image. The positions of these atomic columns are taken to be the average position of the pixels in each of these areas. The sizes and ellipticities of the atomic columns are determined by collecting the pixel positions into an $n \times 2$ matrix $\mathbf{P}$, then calculating the eigenvalues of the covariance matrix of $\mathbf{P}$ centered at the origin. The sizes of the atomic columns are the magnitudes of the eigenvalues, the ellipticities are their ratios. Local lattice parameter values are calculated as the distance between adjacent atomic columns.

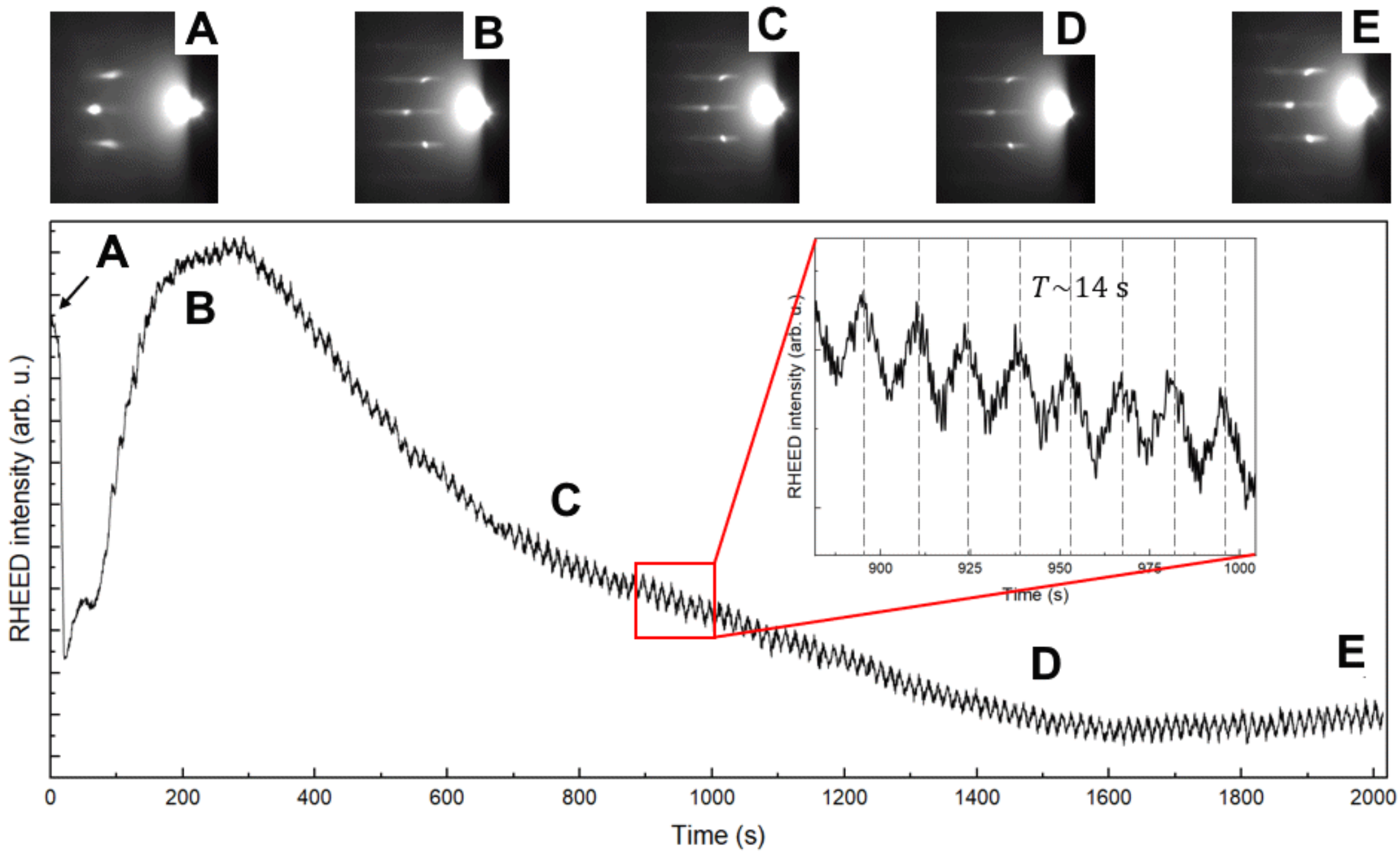

Figure S2: Reflection high-energy electron diffraction (RHEED). a-e) RHEED pattern snapshots collected at different points during the PLD growth of WO_3 films on YAO(001). Bottom panel: RHEED intensity over time. The snapshots at different stages of the growth and the clear intensity oscillations indicate ideal two-dimensional layer-by-layer growth. The images are presented in grayscale, with black and white contrast indicating minimum and maximum intensity. The large panel shows RHEED intensity over time with an approximate 14 s/osc. oscillation period, corresponding to a growth rate of 0.071 monolayer/s.

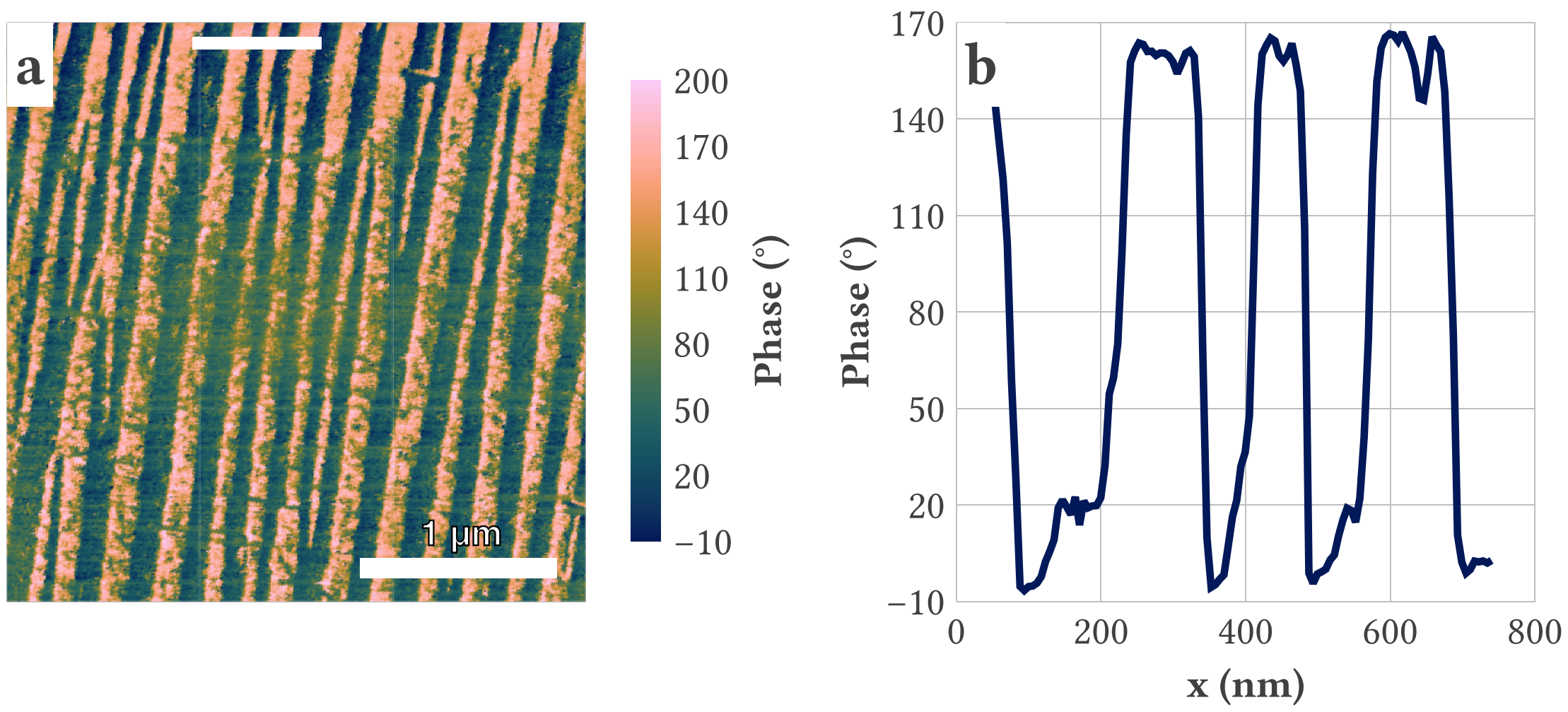

Figure S3: Lateral PFM profile. (a) Lateral PFM phase image measured using the same geometry as Fig. 1a, showing the stripe domain pattern. The white line indicates the location from which the profile in (b) is taken. (b) Profile across the stripe domains, showing 180° domain contrast.

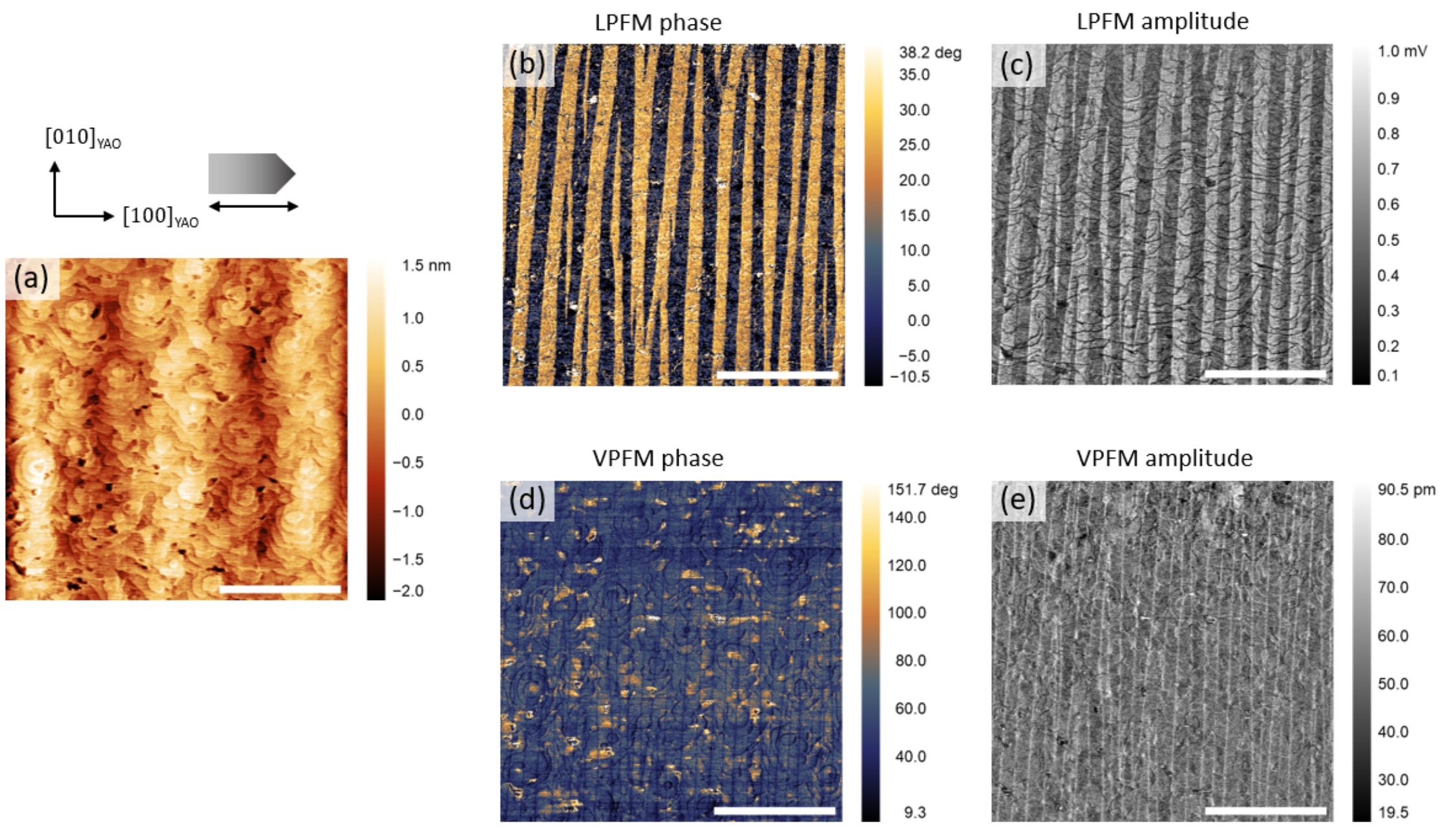

Figure S4: Lateral and vertical PFM images obtained on a 157 nm-thick WO_3 film with the long axis of the cantilever oriented parallel to the [100] axis of the substrate. (a) Topography, (b) Lateral phase, (c) Lateral amplitude, (d) Vertical phase, (e) Vertical amplitude. Note the slightly enhanced vertical PFM amplitude near the stripe domain walls, likely related to a local flexoelectricity-induced vertical polarization component. The scale bar represents 1 µm.

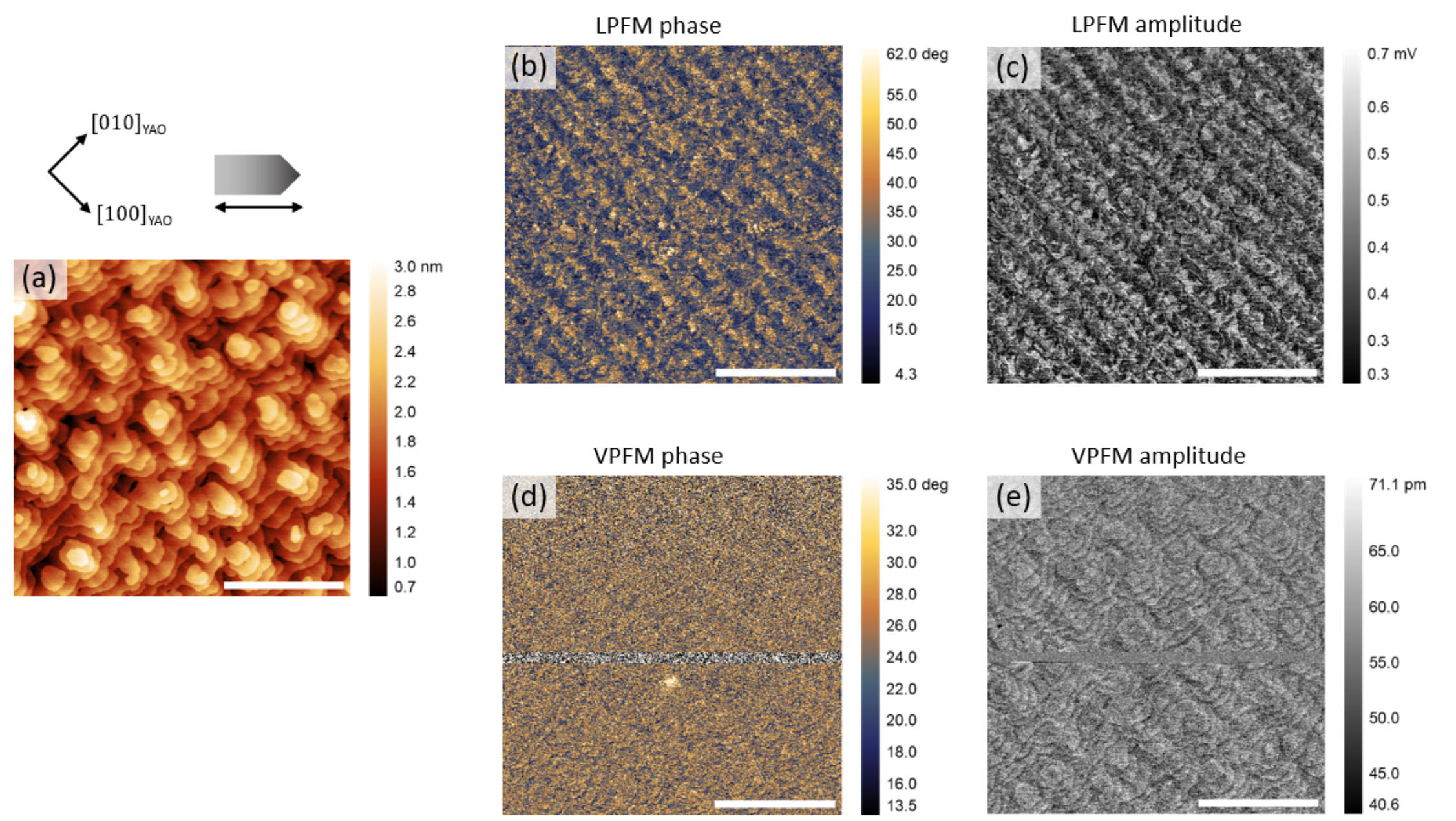

Figure S5: Lateral and vertical PFM images obtained on a 157 nm-thick WO_3 film with the long axis of the cantilever oriented at a 45° angle to the [100] axis of the substrate. (a) Topography, (b) Lateral phase, (c) Lateral amplitude, (d) Vertical phase, (e) Vertical amplitude. The observed lateral piezoresponse corresponds to a combination of stripe (Fig. S4) and mosaic domains (Fig. S6). The scale bar represents 1 µm.

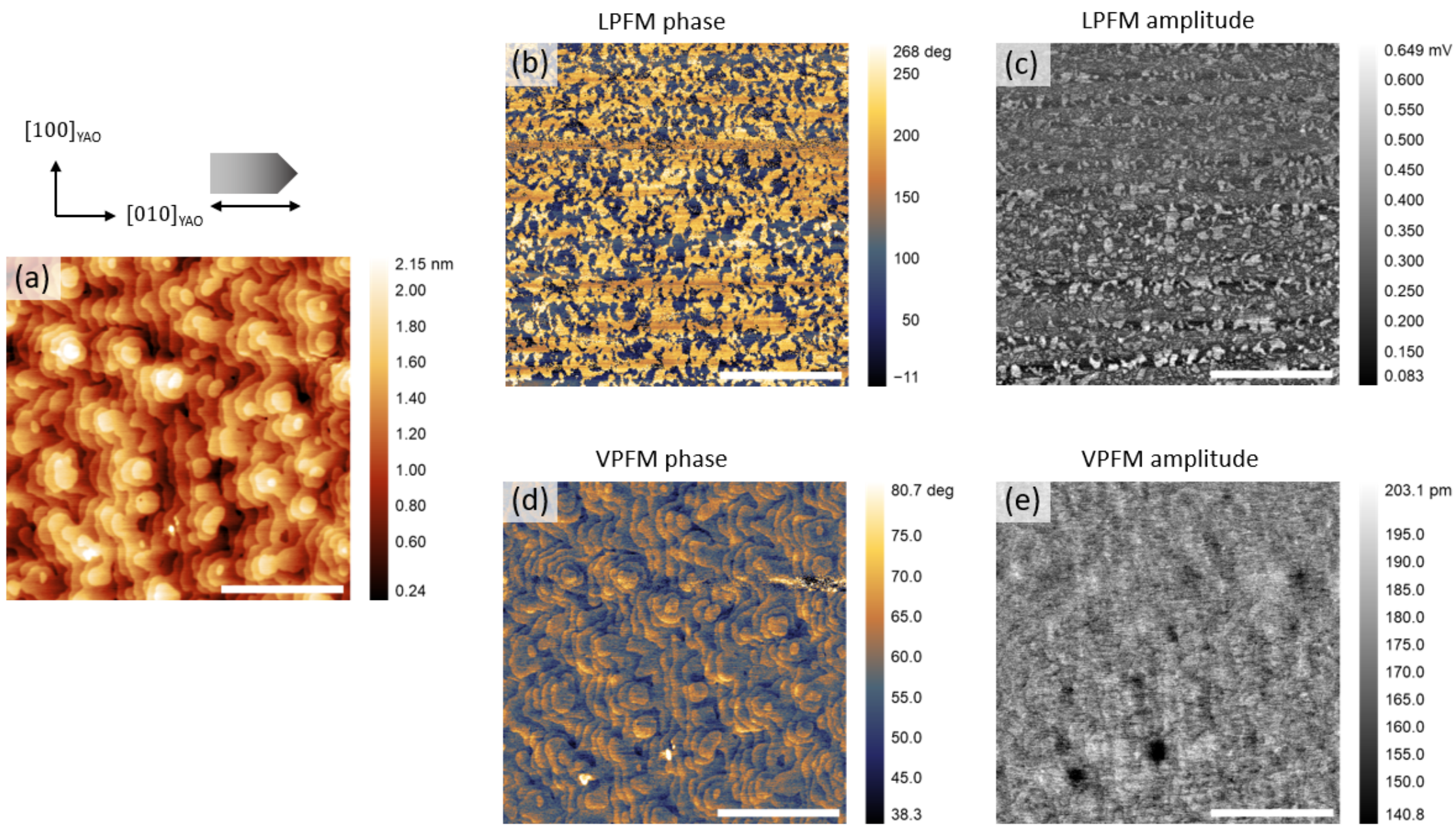

Figure S6: Lateral and vertical PFM images obtained on a 157 nm-thick WO_3 film with the long axis of the cantilever oriented parallel to the [010] axis of the substrate. (a) Topography, (b) Lateral phase, (c) Lateral amplitude, (d) Vertical phase, (e) Vertical amplitude. The scale bar represents 1 µm.

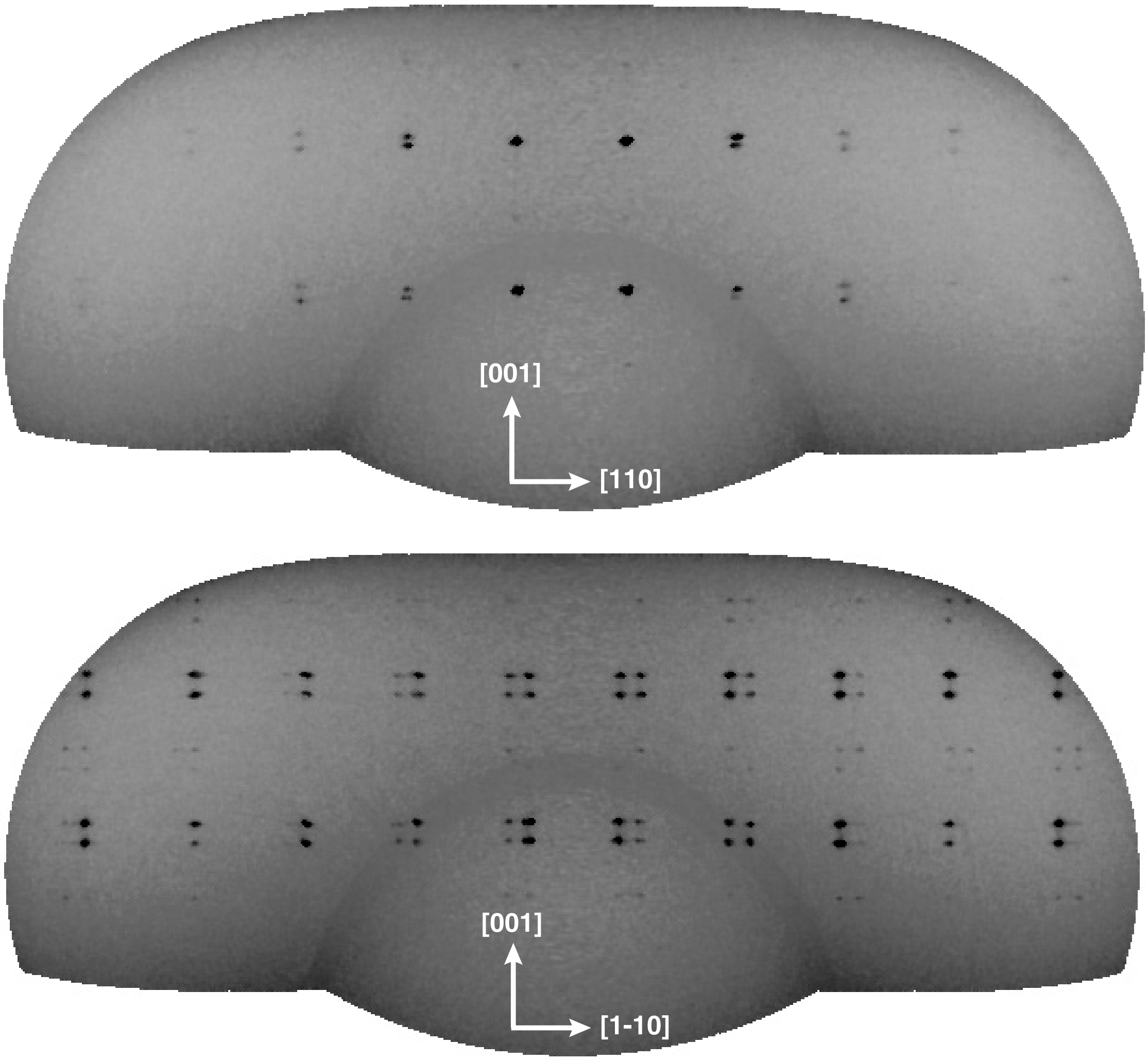

Figure S7: Reconstructed reciprocal lattice planes. Seen along the $[1\bar{1}0]$ (top panel) and [110] (bottom panel). These planes correspond to $\mathrm{n} = 4$ in Fig. 2a,b. The images are presented in grayscale with white and black representing minimum and maximum intensity, respectively.

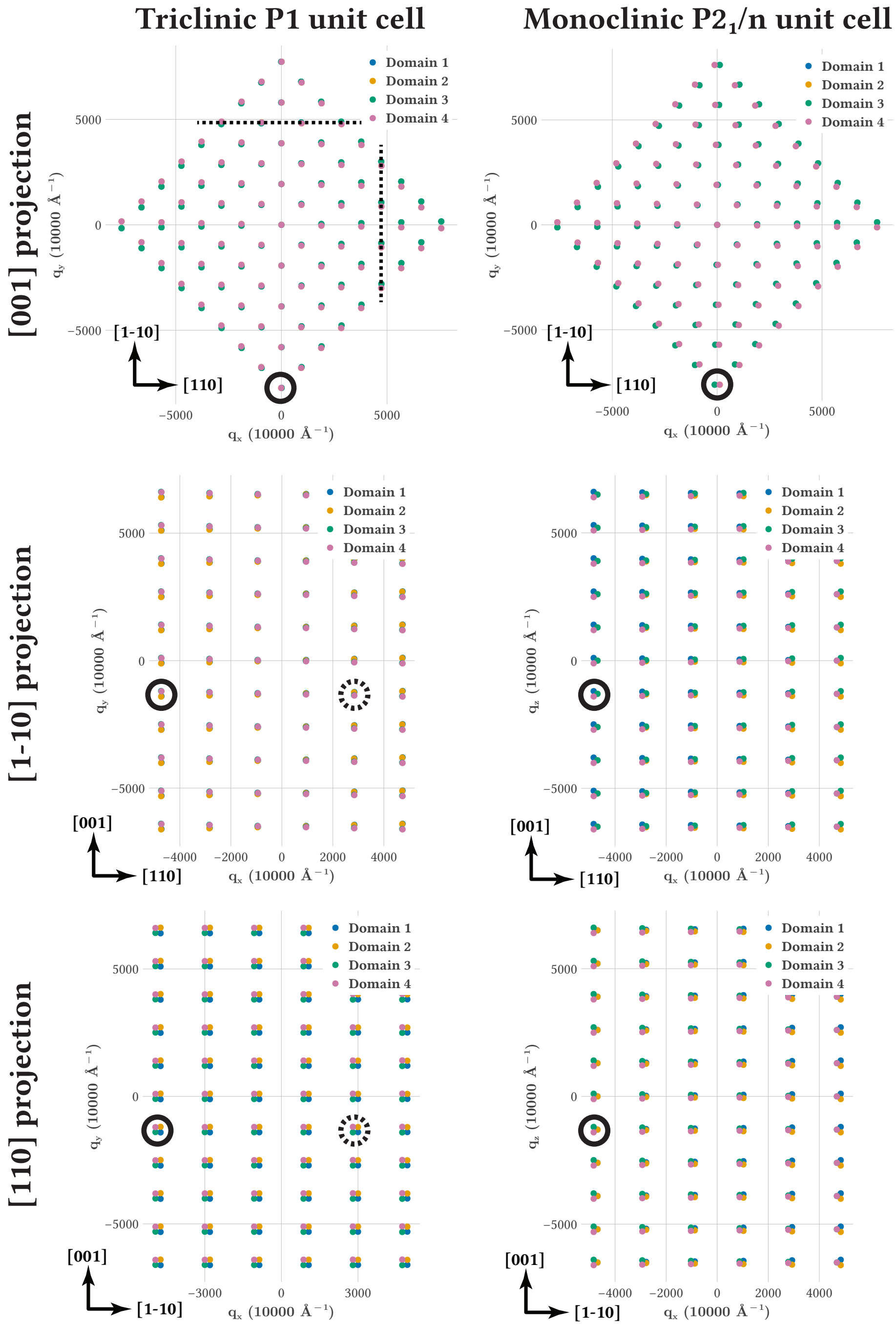

Figure S8: Simulated diffraction patterns of the triclinic phase and monoclinic phases. Left panels: Triclinic phase with four domains, as found in our films. Right panels: equivalent arrangement of monoclinic domains. Solid circles indicate clearly identifyable differences in the symmetry of the diffraction patterns in each of the three directions: [001] (top), $[1\bar{1}0]$ (middle) and [110] (bottom). The planes shown in the middle and bottom plots are indicated in the top plot by dotted lines. The dotted circles show examples of the two-fold and four-fold splitting observed in Fig. 2a,b. The image series presented in Fig. 2 represent reciprocal lattice planes of increasing order as viewed along the direction perpendicular to the planes plotted here,*i.e.* the projection directions $[1\bar{1}0]$ and [110], respectively (see also Fig. S9).

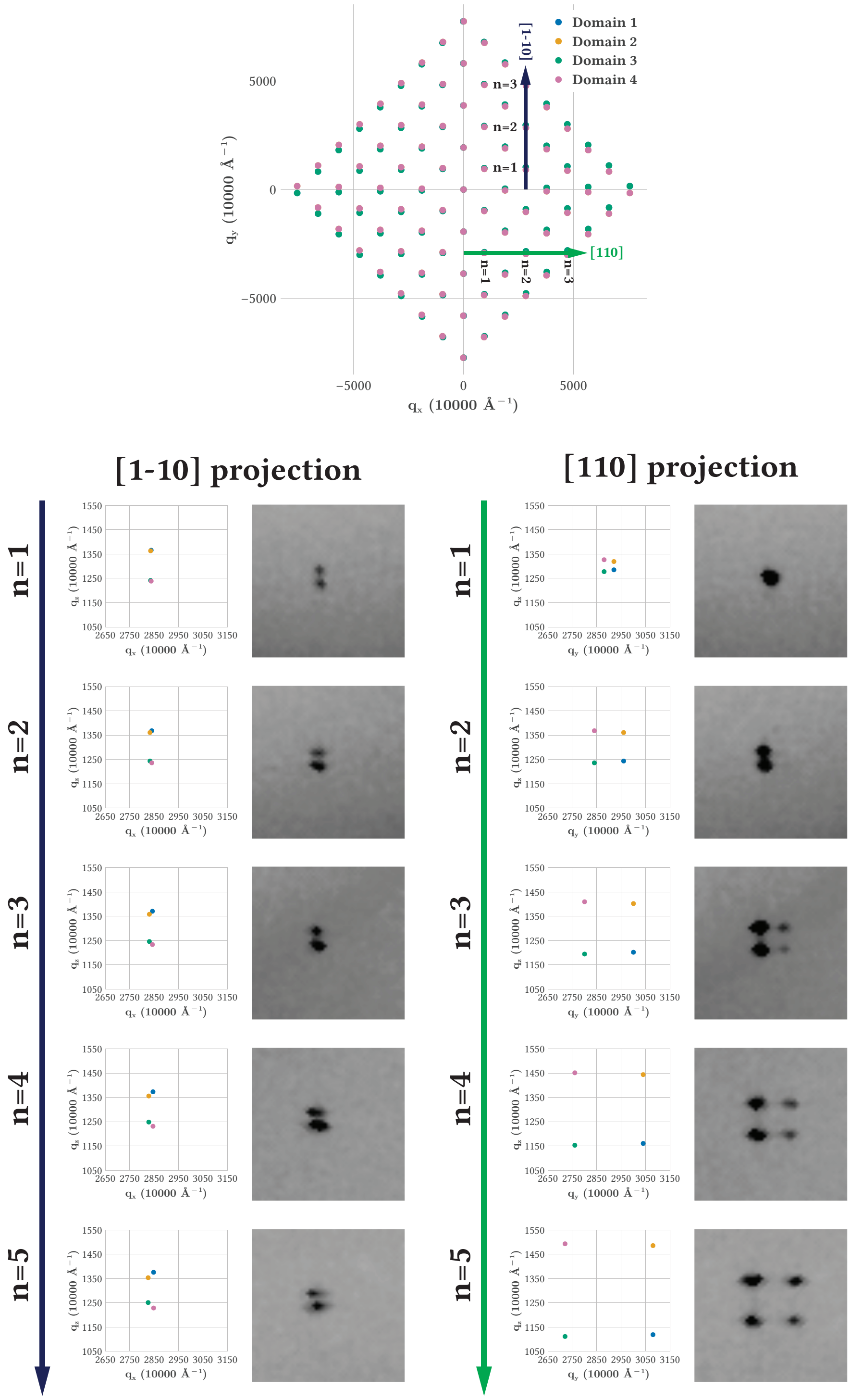

Figure S9: Simulated reciprocal lattice planes compared to experimental data. The experimental images are equivalent to the image series in Fig. 2a,b, showing the distinct two-fold splitting which does not increase with diffraction order along the $[1\bar{1}0]$ direction (left), as well as the four-fold splitting along [110] which does (right). The small splitting between domains 1/3 and 2/4 visible along $[1\bar{1}0]$ cannot be resolved within the resolution of this measurement. The experimental images are presented in grayscale with white and black representing minimum and maximum intensity, respectively.

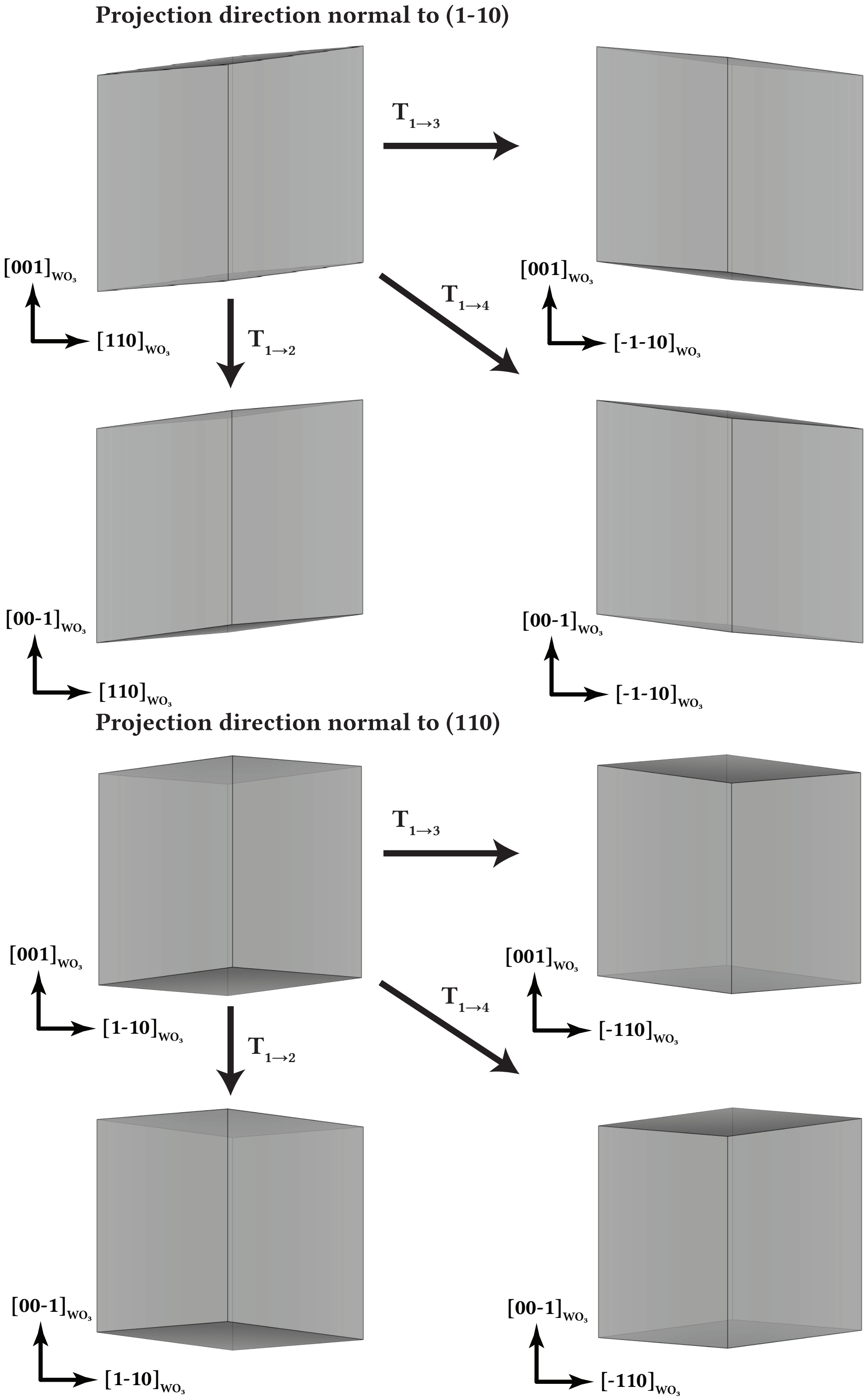

Figure S10: Relative orientations of the four structural domains with respect to each other. Viewed along the real-space normals of the (1-10) and (110) planes. The arrows indicate the twinning operations as defined in Supplementary Note S2.

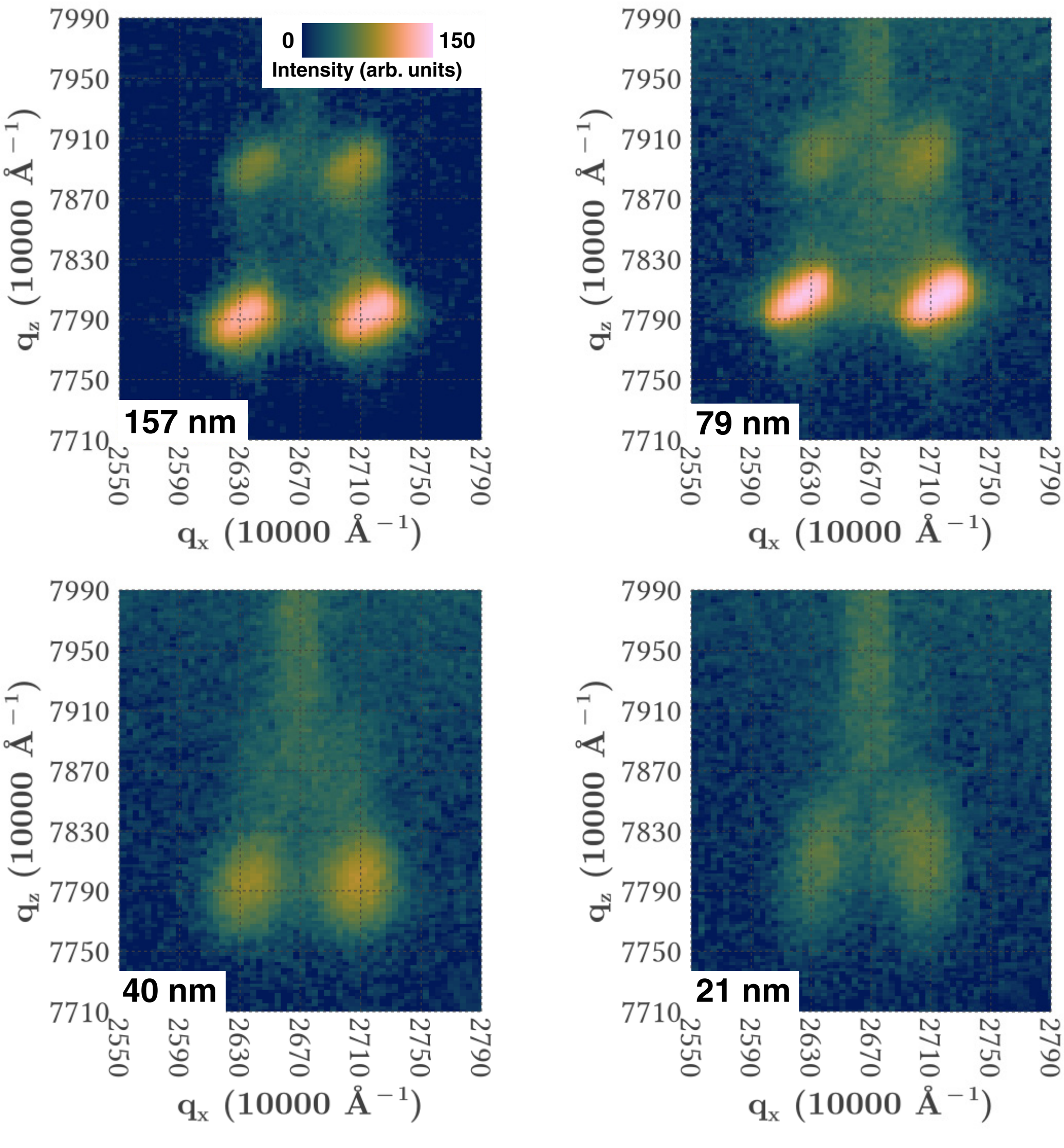

Figure S11: Reciprocal space maps at different thicknesses. The RSMs are collected close to the pseudocubic (103) reflections of the YAO substrate of WO_3 films with thicknesses between 157 nm (top left) and 21 nm (bottom right). The triclinic peak splitting persists across the full thickness range.

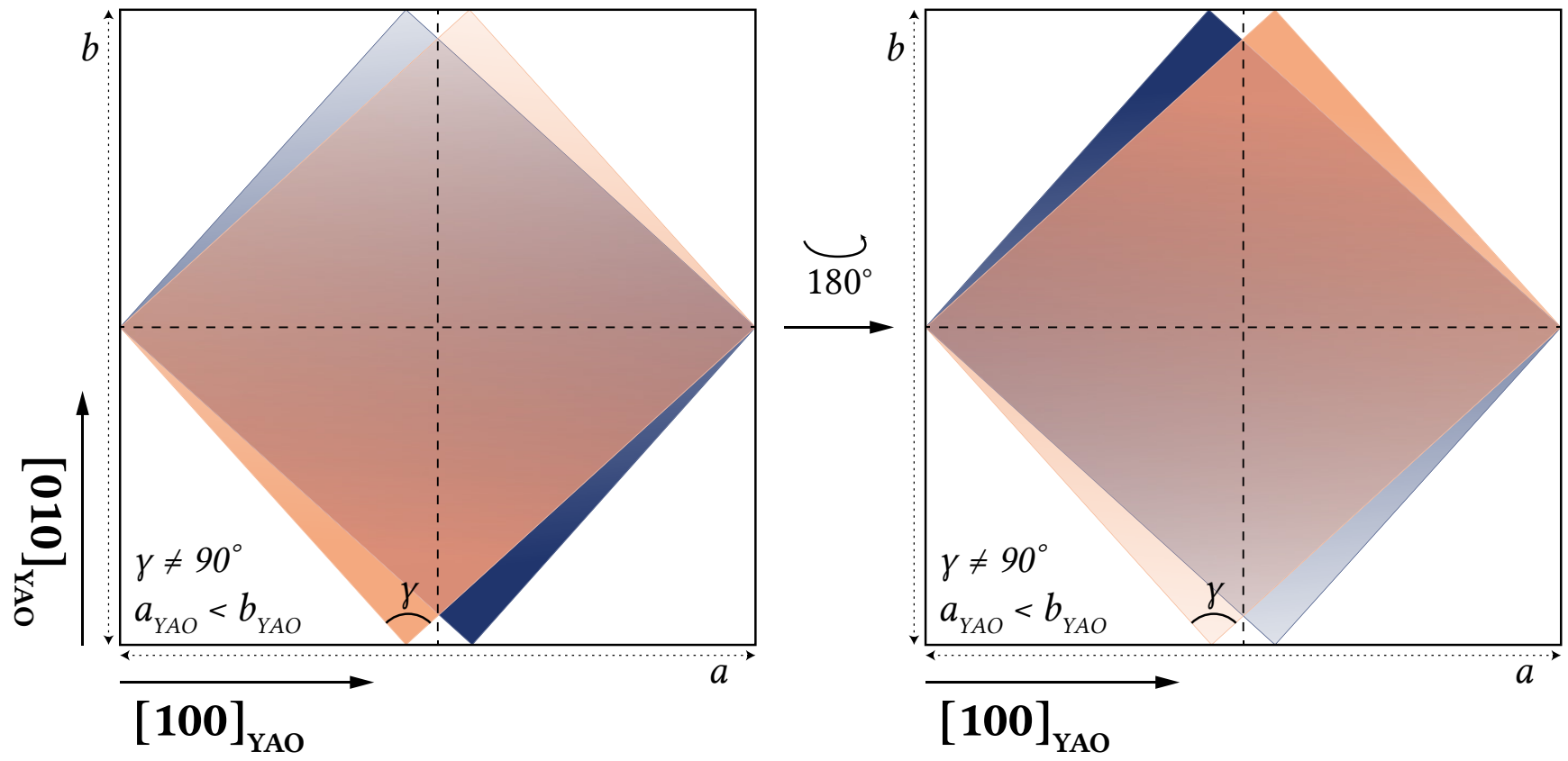

Figure S12: Diagram of the four structural domains. The diagram shows the relation between the four structural domains in the WO_3 film and the $YAlO_3$ substrate. The color gradient indicates an out-of-plane tilt of the film unit cell around the $[100]_{YAO}$ axis. The first two domains (left panel, identical to Fig. 2d) are related to each other by a 180° rotation about the [100] axis of the substrate and to the other two domains (right panel) by a rotation about either the [001] or the [010] axis of the substrate.

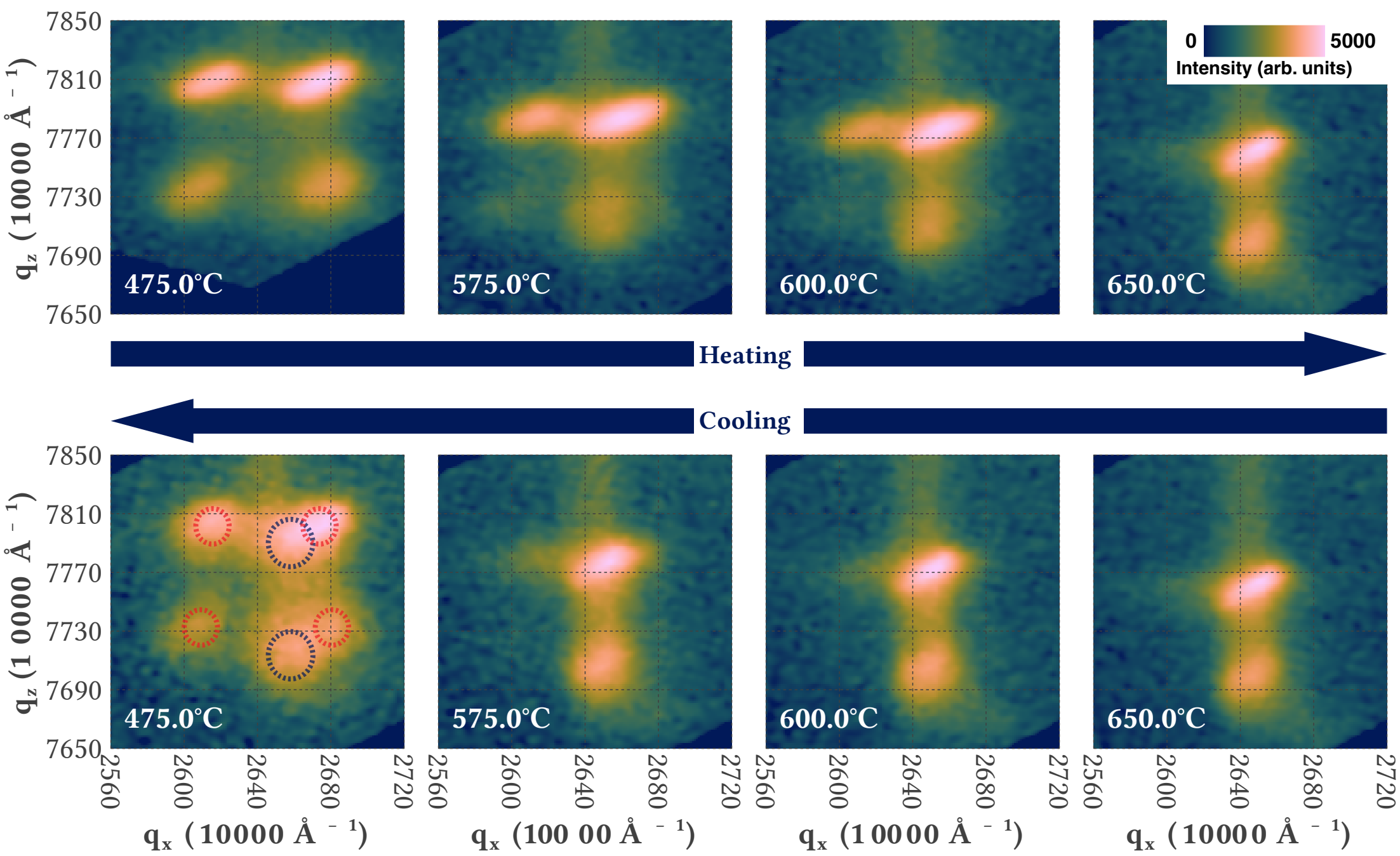

Figure S13: Reciprocal space map of the pseudo-cubic (103) reflections of the WO_3 film at different temperatures through the phase transition. Top panels: RSMs on heating between 475 °C and 650 °C, bottom panels: RMSs at the same temperatures on cooling. The dotted circles in the RSM at 475 °C on cooling indicate the positions of the diffraction spots of the low-temperature triclinic phase in red and of the high-temperature phase in blue, showing clear phase coexistence (*cf.* the same temperature on heating).

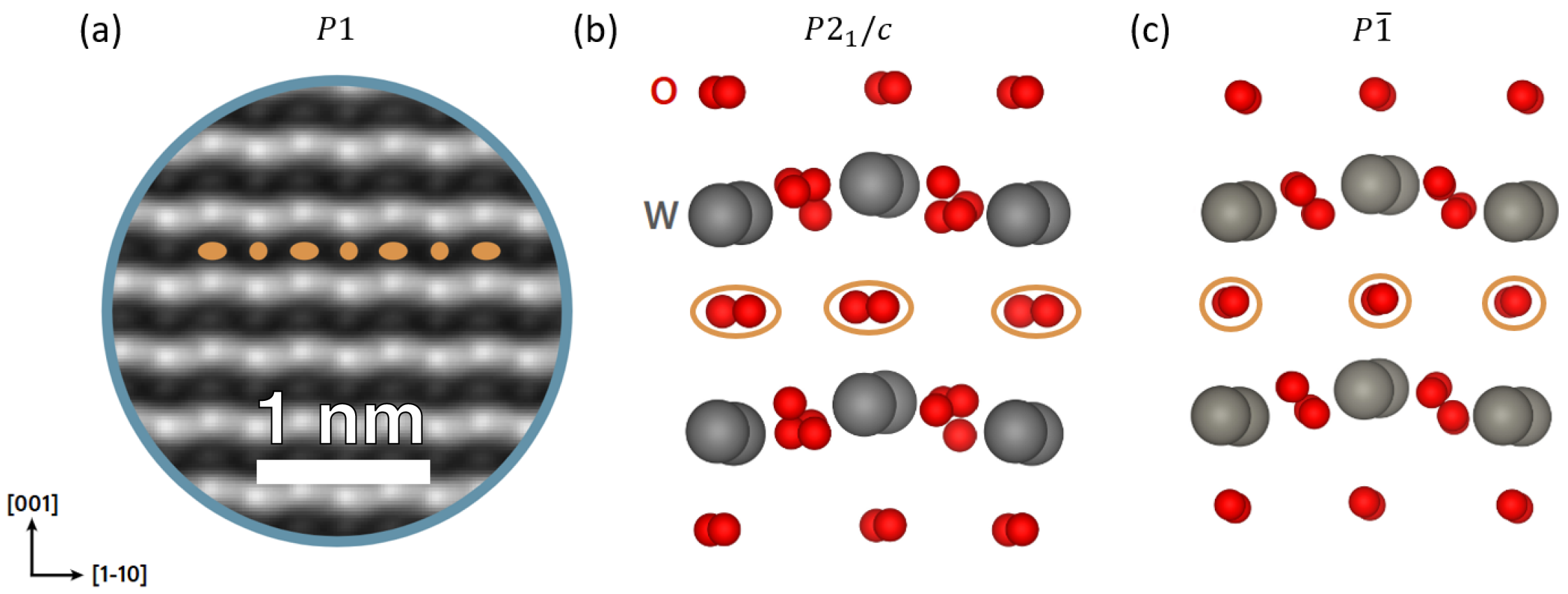

Figure S14: Comparison of experimental STEM image with structural models. (a) Experimental STEM image, (b,c) unit cells of the (b) *P2₁/n* (octahedral tilting pattern $a^-b^+c^-$) and (c) *P-1* (octahedral tilting pattern $a^-b^-c^-$) structures found in bulk WO_3 in the (110) zone. Note that all atomic columns of oxygen in the V_AO sublayers are identical, unlike in our films. The iDPC-STEM image is presented in grayscale, black and white representing minimum and maximum projected electrostatic potential, respectively.

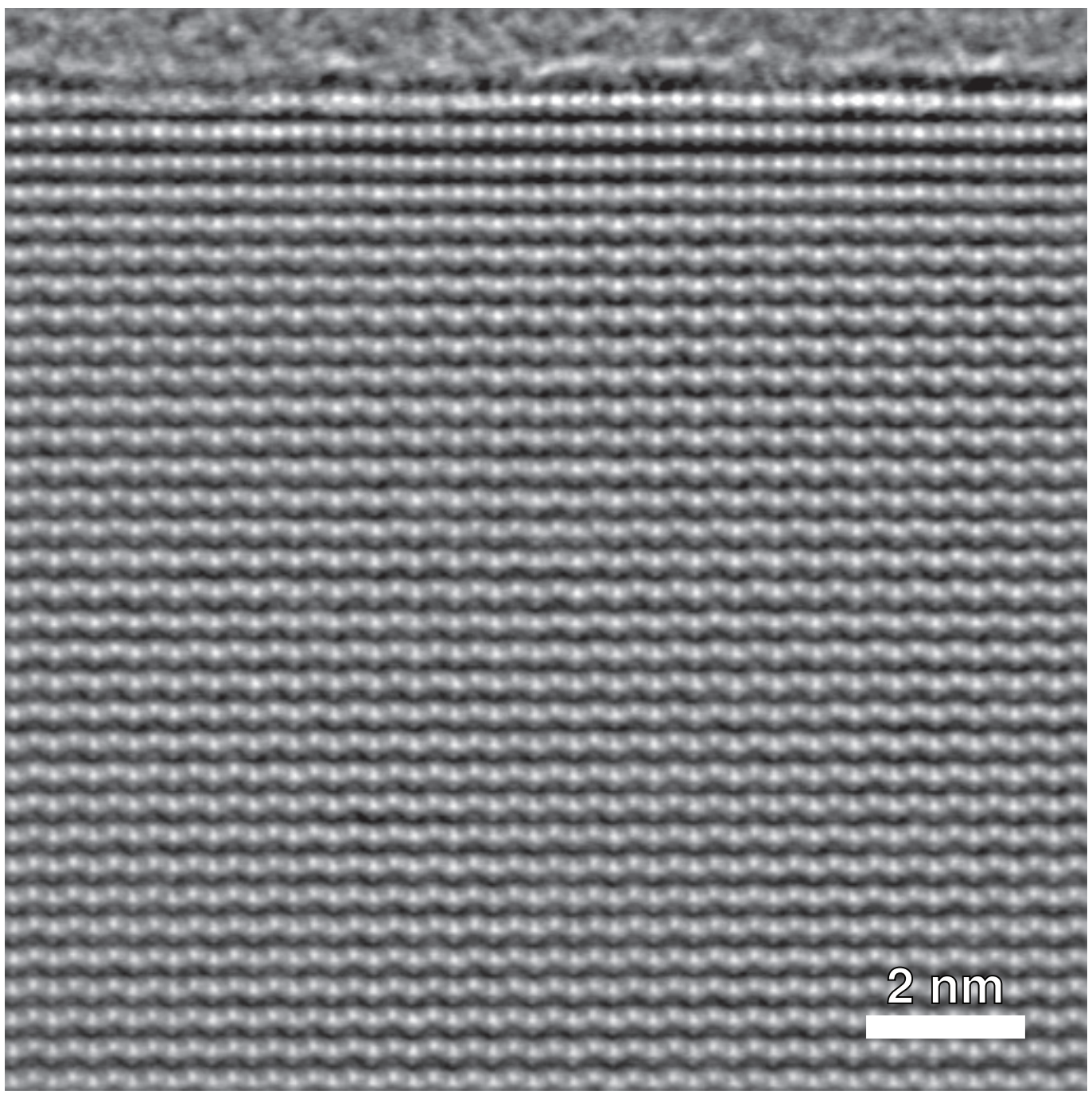

Figure S15: iDPC-STEM image close to the film surface. The image shows a change of the structure similar to that seen close to the domain walls. The image is presented in grayscale, black and white representing minimum and maximum projected electrostatic potential, respectively.

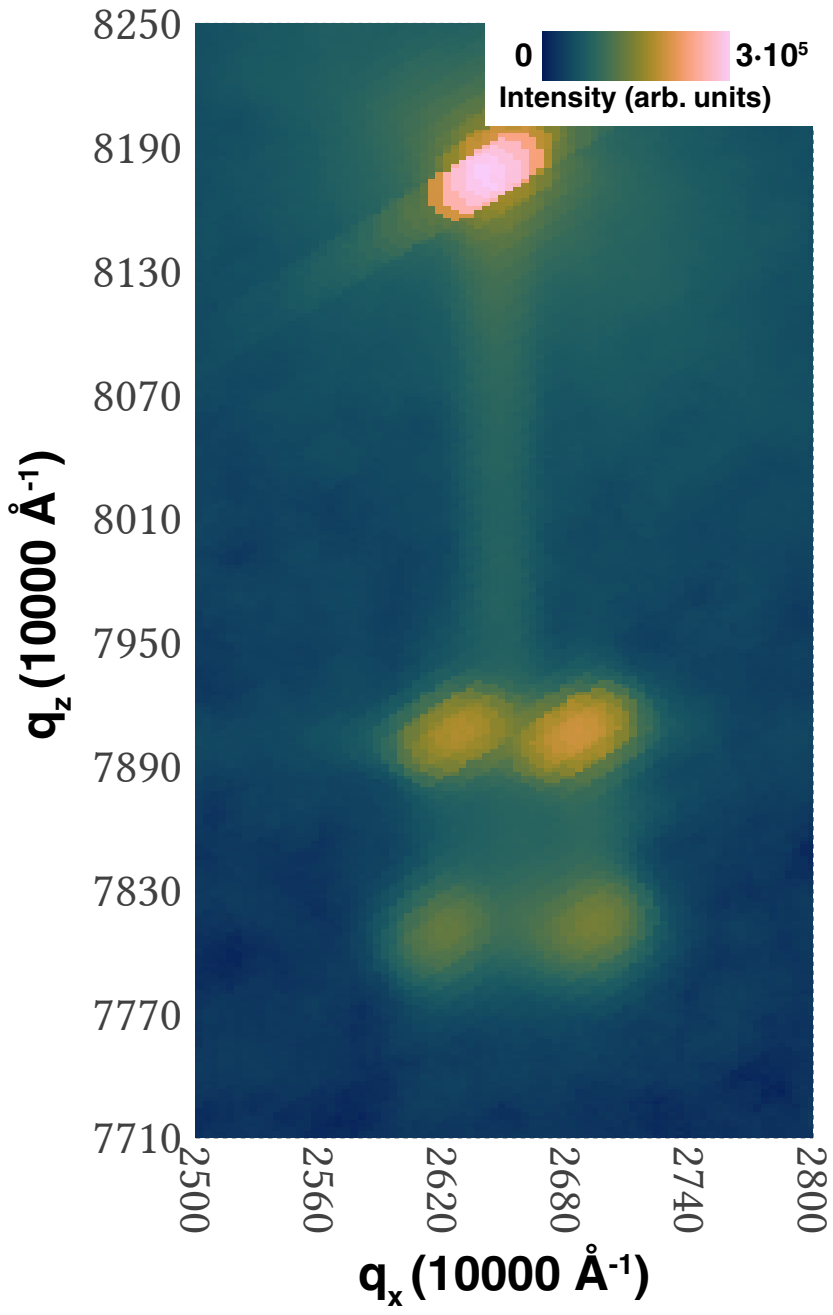

Figure S16: Reciprocal space map of the pseudo-cubic (103) reflection of an approximately 392 nm thick WO_3 film, still showing triclinic peak splitting.

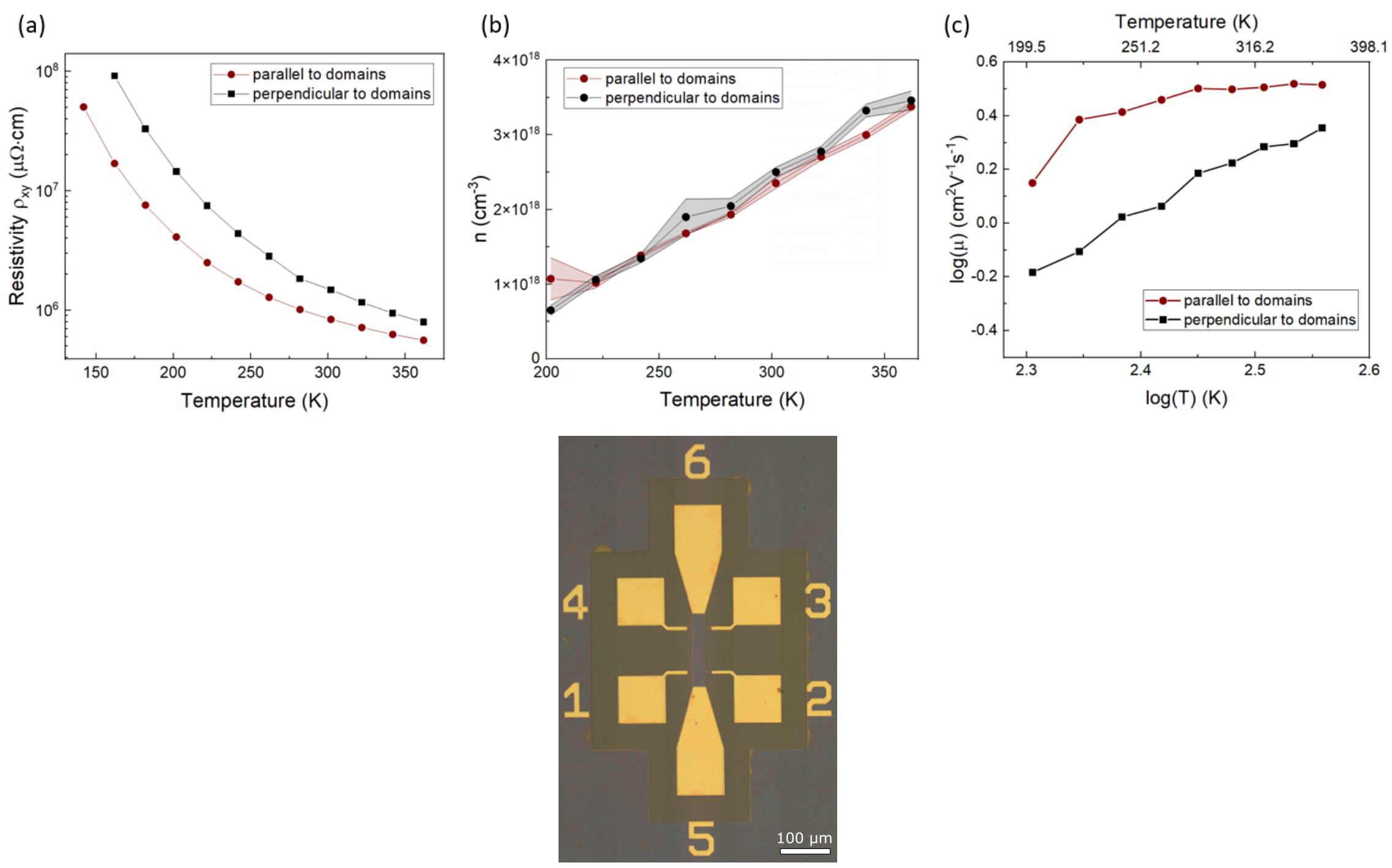

Figure S17: a) Resistivity, b) carrier concentration and c) mobility extracted from Hall bar measurements. Bottom panel: Optical microscopy image of the Hall bar geometry used for these measurements. The shaded region in (b) represents the standard error in the data points.

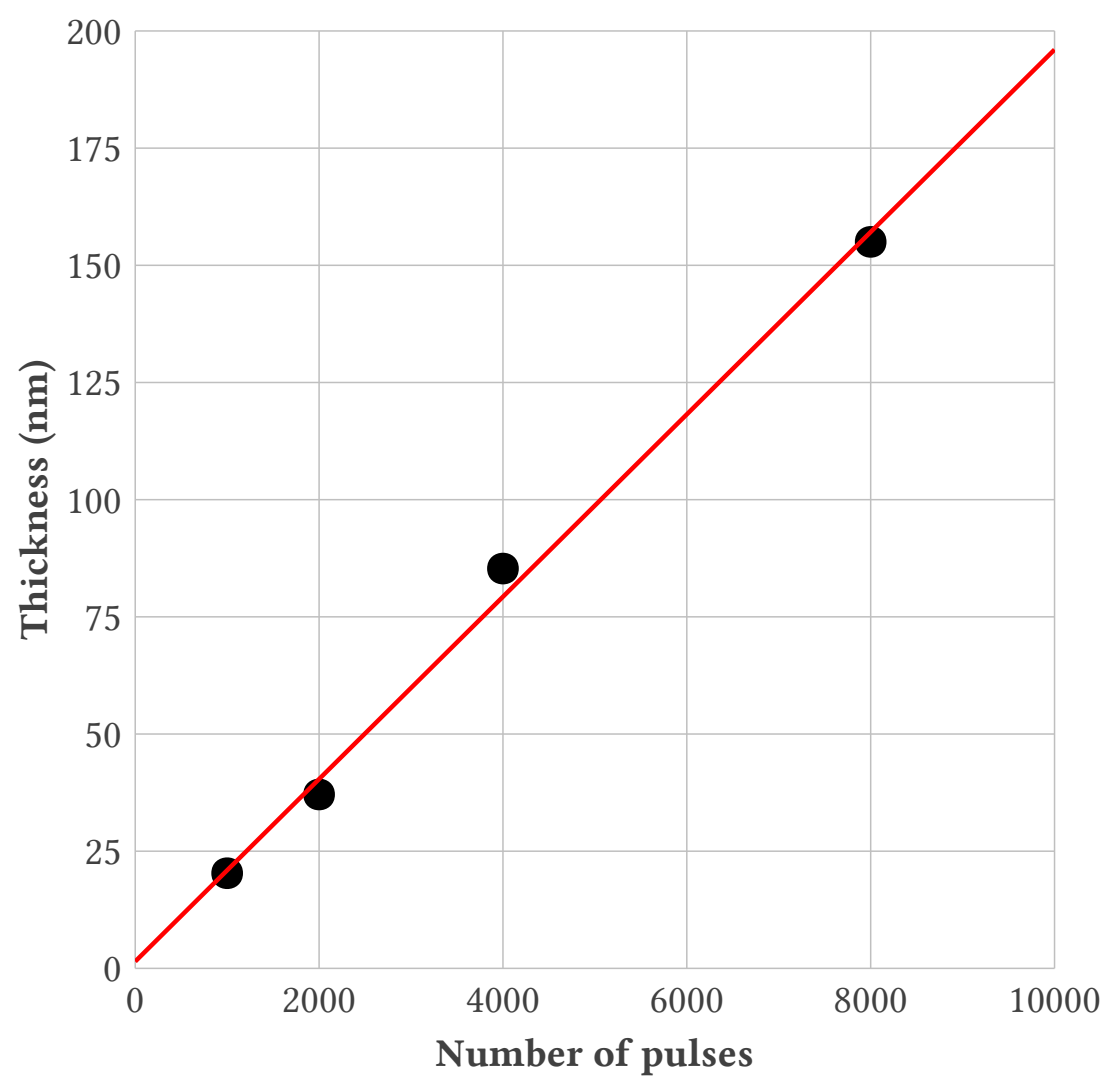

Figure S18: Thickness of the WO_3 films grown by pulsed layer deposition on (001)$YAlO_3$ substrates, as a function of the number of laser pulses. The data points were measured from the spacing of Laue oscillations in Fig. 2c. The thickness values given in the main text were obtained from the linear fit shown in red.

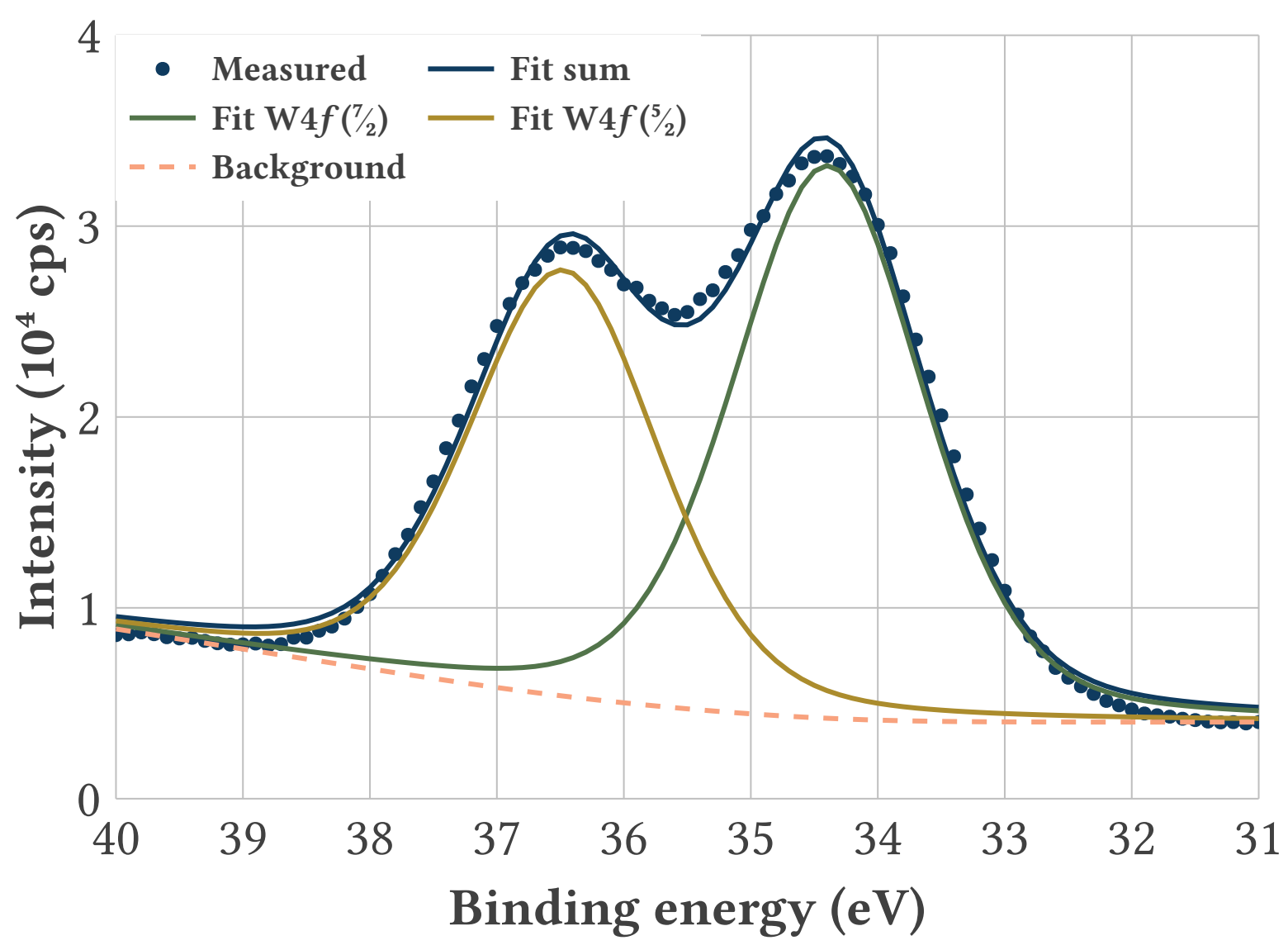

Figure S19: X-ray photoelectron spectrum of the W4f peak showing a single valence state for tungsten. The lack of W^{5+} implies that the film is near-stoichiometric.

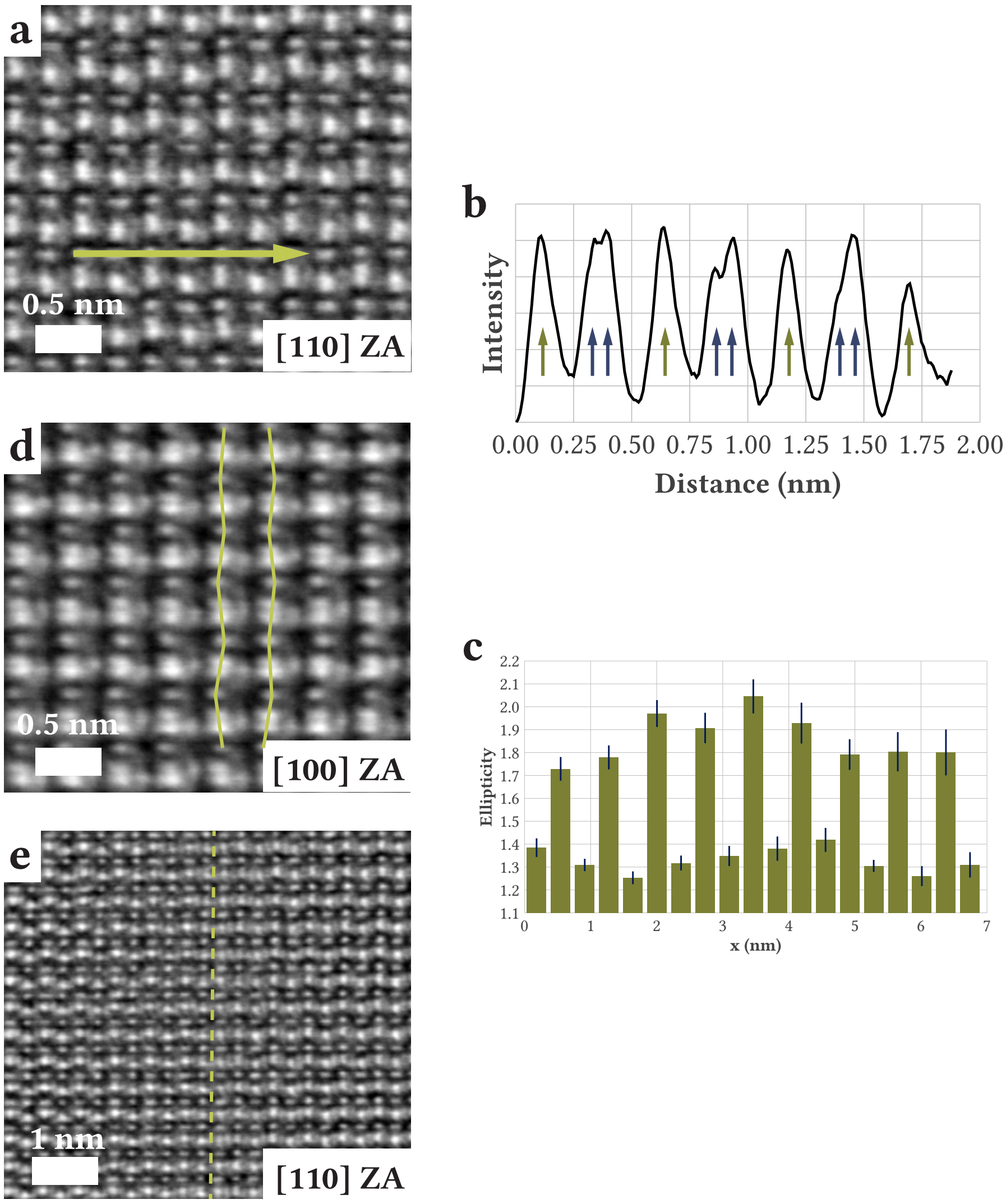

Figure S20: a) Additional non-drift-corrected iDPC-STEM image of a thin (50 – 70 nm) lamella. The image still shows the wide-narrow pattern of oxygen atomic column ellipticities. b) Line profile through the oxygen atomic columns, corresponding to the green arrow in (a). The wide-narrow pattern again visible. The broad columns show a slight peak splitting, as indicated by the blue double arrows. This peak splitting is indicative of anti-phase octahedral tilts being the origin of the broadening of alternating oxygen atomic columns. c) Vertically integrated profile of ellipticities of the oxygen atomic columns extracted from a larger iDPC-STEM image of a thin lamella. Each bar represents one vertical column in the image. The wide-narrow pattern is evident. The error bars represent the standard error of the averaged values of the ellipticity. d) iDPC-STEM image in the [100] zone of a thin lamella. The green vertical traces show the antiphase octahedral tilting the the out-of-plane direction. e) Additional iDPC-STEM image collected from a thin lamella showing a domain wall. The suppression of W atomic column modulation and associated 180° rotation about the projection direction (equivalent to two of the structural twins seen in the x-ray diffraction results) are visible. The exact position of the domain wall is indicated by a dashed line. The iDPC-STEM images are presented in grayscale, black and white representing minimum and maximum projected electrostatic potential, respectively.

References

1. Yun, S. *et al.* Ferroelastic twin structures in epitaxial WO_3 thin films. *Appl. Phys. Lett.* **107** (2015).

2. Yun, S. *et al.* Flexopiezoelectricity at ferroelastic domain walls in WO_3 films. *Nat. Commun.* **11,** 4898 (2020).

3. Marcus, P. M. & Jona, F. Strains in epitaxial films: The general case. *Phys. Rev. B* **51,** 5263–5268 (8 Feb. 1995).

4. Rosen, C., Banks, E. & Post, B. The thermal expansion and phase transitions of WO_3. *Acta Crystallogr.* **9,** 475–476 (June 1956).

5. Chaix-Pluchery, O., Chenevier, B. & Robles, J. J. Anisotropy of thermal expansion in $YAlO_3$ and $NdGaO_3$. *Appl. Phys. Lett.* **86,** 251911 (June 2005).

6. Niblack, W. *An introduction to digital image processing* 215 (Prentice-Hall International, 1986).
