# Supplementary material for: Polarity and anti-distortive polarons in WO3 through epitaxial shear strain
Source: Nat Commun. 2026 Jul 13;17:8593. doi: 10.1038/s41467-026-75049-x (PMC13486495; doi:10.1038/s41467-026-75049-x)
Supplement: Supplementary file 2 [file 41467_2026_75049_MOESM2_ESM.pdf]

Supplementary video for: Polarity and anti-distortive polarons in WO_3 through epitaxial shear strain

Ewout van der Veer‡ * [1,2,3], Martin F. Sarott‡ [1,4], Jack T. Eckstein [5,6], Stijn Feringa [1], Dennis van der Veen [1], Johanna van Gent González [1], Majid Ahmadi [1,4], Horatio R. J. Cox [1,4], Ellen M. Kiens [7], Gertjan Koster [7], Bart J. Kooi [1,4], Michael A. Carpenter [5], Ekhard K. H. Salje† [5], and Beatriz Noheda * [1,4]

[1]Zernike Institute for Advanced Materials, University of Groningen, Groningen, The Netherlands

[2]Faculty of Physics, University of Duisburg-Essen, Duisburg, Germany

[3]Research Center Future Energy Materials and Systems (RC FEMS), Research Alliance Ruhr, Bochum, Germany

[4]Groningen Cognitive Systems and Materials Center (CogniGron), University of Groningen, Groningen, The Netherlands

[5]Department of Earth Sciences, University of Cambridge, Cambridge, United Kingdom

[6]Center for Nanophase Materials Sciences, Oak Ridge National Laboratory, Oak Ridge, Tennessee, USA

[7]MESA+ Institute for Nanotechnology, University of Twente, Enschede, The Netherlands

* Corresponding authors. Email: ewout.vanderveer@uni-due.de (E.vdV.), b.noheda@rug.nl (B.N.)

‡ These authors contributed equally to this work.

Supplementary Video S1 Video of the phase transition

Animated temperature-dependent x-ray diffraction pattern of the (226) diffraction peaks of the WO_3 film as the sample is heated through the phase transition into the high-temperature phase and subsequently cooled back down to the triclinic phase, showing that the transition is reversible. Phase coexistence can be observed in the intermediate temperature region. The color scale of the video is the same as that in Supplementary Fig. S13.
