# Supplementary material for: Polarity and anti-distortive polarons in WO3 through epitaxial shear strain
Source: Nat Commun. 2026 Jul 13;17:8593. doi: 10.1038/s41467-026-75049-x (PMC13486495; doi:10.1038/s41467-026-75049-x)
Supplement: Supplementary file 3 [file 41467_2026_75049_MOESM4_ESM.pdf]

nature portfolio

Peer Review File

Polarity and anti-distortive polarons in WO_3 through epitaxial shear strain

Corresponding Author: Dr Ewout van der Veer

This file contains all reviewer reports in order by version, followed by all author rebuttals in order by version.

Version 0:

Reviewer comments:

Reviewer #1

(Remarks to the Author)
Ewout van der Veer et al. report that a new polar phase of WO3 is stabilized on a YAlO3(001) substrate through epitaxial shear strain, leading to polaronic conduction along domain walls where the distortive atomic mode is significantly suppressed. Strain engineering is a well-established strategy for tuning ferroic properties (magnetization and electrical polarization), electronic conduction, superconductivity, and for stabilizing metastable phases. While most prior studies have focused on longitudinal tensile or compressive strain originating from lattice mismatch, the authors emphasize the role of epitaxial shear strain in stabilizing a metastable polar phase. WO3 is indeed a suitable platform for such investigation, as it exhibits diverse structural morphologies depending on temperature and substrate-induced strain. Moreover, phenomena such as bulk polarons, domain-wall conduction, and even domain-wall superconductivity have been reported in its low-temperature polar structures. By combining PFM, XRD, STEM, and transport measurements, the authors aim to demonstrate that epitaxial shear strain drives the emergence of a polar phase in WO3.

The study is intriguing and potentially impactful. However, the manuscript requires clearer presentation of the experimental observations and stronger justification for the proposed shear-strain-induced phase stabilization and polaronic behavior. I outline several points below that I believe will help improve the clarity and rigor of the manuscript.

1) Clarification of PFM Phase Contrast (Figure 1b)
In Figure 1b, the phase difference between adjacent in-plane polarization domains appears to be approximately 40°, rather than the expected 180° for antiparallel polarization directions. Could the authors clarify why a 180° phase contrast is not observed in this measurement? Additional explanation regarding the PFM phase response in this system would be helpful.

2) Interpretation of XRD Results
The XRD analysis is extensive, yet several aspects remain difficult to follow without additional explanation. Addressing the points below would significantly improve the clarity of the structural interpretation.

2-1) Diffraction Peak Separation in Figure 2b
In Figure 2b and line 146, the two diffraction peaks along the [100]_YAO direction maintain the same separation as the diffraction order increases. Could the authors clarify why these peaks do not exhibit the distinct triclinic domain behavior observed in Figure 1a?

2-2) Methodology Behind Table 1 (Crystallographic Data)
Table 1 is central to distinguishing the proposed polar phase from previously reported WO3 structures. However, the analytical procedure and statistical basis for extracting the crystallographic parameters are not sufficiently described. Providing details on the refinement method, fitting constraints, and error analysis would greatly strengthen the structural argument and clarify the triclinic peak splitting discussed in line 150.

2-3) Explicit Assignment of Diffraction Peaks to Triclinic Domains
Although lines 164–169 and Figure 2d describe the triclinic domains, the interpretation remains difficult without explicit one-to-one assignments between diffraction peaks and real-space domain variants. While the splitting along q_x in Figure 2e can be attributed to the fact that gamma deviates from 90°, the manuscript does not explain the additional splitting along q_z. A more detailed description of the triclinic domain geometry, supported by Figure S7 and explicit peak-to-domain mapping,

would greatly aid reader comprehension.

2-4) Quantitative Description of Epitaxial Shear Strain
The non-orthogonal base angle of $YAlO_3$ (91.5°) is presented as the origin of the epitaxial shear strain that stabilizes the polar phase (lines 122, 194). However, the discussion remains qualitative. A quantitative analysis of the shear strain imposed on WO_3—its magnitude, tensor components, and orientation—would significantly strengthen the argument and highlight the novelty of the shear-strain-driven mechanism.

2-5) Temperature-Dependent Shear Strain and Structural Evolution
If temperature-dependent shear strain values for $YAlO_3$(001) are available from literature or previous experiments, correlating them with the temperature-dependent structural evolution of WO_3 (Figure 2f) would deepen the discussion and reinforce the proposed mechanism.

3) Directional Notations in Figure 4
In Figures 4a–c, adding directional labels would prevent potential misinterpretation that the polarization direction rotates with increasing film thickness. Clear axis or crystallographic direction annotations would improve readability.

4) Claim of "First Observation" of Anti-Distortive Polarons
The manuscript states that this work presents the first observation of an anti-distortive polaron (citing H. Hassani et al., Nat. Commun. 16, 1688 (2025)). However, among the four papers citing that work, an experimental study on WO_3 has already been published (G. Lee et al., Small 21, e04162 (2025)). The authors should clarify whether the present manuscript provides additional or more direct evidence that justifies emphasizing the "first" observation, or revise the claim accordingly.

5) Origin of Domain-Wall Conduction and the Definition of Anti-Distortive Polarons
The manuscript attributes the enhanced domain-wall conduction to anti-distortive polarons. However, the STEM images show that the domain-wall region appears undistorted even without charge transport, suggesting that the structure may already adopt an anti-distortive configuration. In this case, can the phenomenon still be classified as a polaron, which conventionally involves lattice distortion induced by charge localization? Alternatively, could the enhanced conduction be explained by the higher structural symmetry and reduced band gap at the domain wall, as suggested by structure-dependent calculations in Hassani et al.? Clarifying this conceptual distinction would strengthen the interpretation.

5-1) Transport Perpendicular to Domain Walls (Figure 5c)
If conduction is dominated by polarons localized along domain walls, it is unclear how this mechanism would contribute to transport measured perpendicular to the stripe walls. In such a configuration, bulk conduction with higher activation energy would be expected to dominate. The authors should clarify whether polaronic transport is indeed relevant in this measurement geometry.

Overall Assessment
The manuscript presents potentially significant findings regarding shear-strain-induced polar phases and domain-wall conduction in WO_3. Addressing the points above—particularly the structural analysis, quantitative strain discussion, and clarification of the polaronic mechanism—would substantially strengthen the manuscript and improve its clarity for readers.

Reviewer #2

(Remarks to the Author)
In the manuscript entitled "Polarity and anti-distortive polarons in WO_3 through epitaxial shear strain", van der Veer et al. reported the stabilization of a new polar phase in epitaxial WO_3 thin films grown on (001)-oriented YAO substrates. The authors determined the structure of this new phase at various scale by a combination of STEM, PFM and XRD. They also revealed via cAFM an enhanced conductivity at the stripe-domain walls featuring a pronounced reduction of the structural distortions. The conduction mechanism was pointed to anti-distortive polarons recently predicted in WO_3, further supported by the electrical transport measurements. Overall, this work is well designed and presented. I recommend this manuscript for publication in Nature Communications, given the following questions and suggestions are addressed.
1) A new polar and piezoelectric phase is stabilized in epitaxial WO_3 thin films. Have the authors performed secondary harmonic generation (SHG) measurements on the strained WO_3 thin films?
2) What's the relationship between Fig. 1(b) and Fig. S2(b)? Why are the two images presented in a mirror symmetric manner?
3) The statements in line 105-106: "the stripe domains reappear in the vertical channel" and line 110-111: "No domain contrast is found in vertical PFM (see Fig. S5)" are confusing, as the experiment details are not clear, such as fast scanning axis and sample orientation. For a broad readership, the authors are advised to schematically illustrate the line 104-106 and the inset in Fig. 1(d) in the manuscript or supplementary file, and to provide more experimental information of Fig. S5.
4) Could the authors perform the domain switching utilizing the mechanical trailing field of the PFM scanning tip?
5) Regarding the macroscopic structural characterization, could the authors clarify "in one of four directions" in line 166-167? The statement in line 174-175 contradicts Fig. S8, as the bottom diffraction spot exists when temperature is above 600 ∘C. Do the bottom two spots disappear or merge in the first step phase transition around 600 ∘C (line 183)? A more clear and consistent description is necessary.
6) Could the authors present in supplementary file the RSM data of WO_3 films with a thickness larger than 250 nm?
7) The iDPC-STEM image quality in Fig. 3 is inadequate to give accurate information on the oxygen octahedral tilt pattern of

the triclinic P1 phase WO3, maybe due to a thick TEM sample, giving large error on the fitting of oxygen atoms and the calculation of their ellipticity. Taking iDPC-STEM on thinner TEM sample will help, or the authors could use ADF or ABF to visualize the oxygen atoms, if high quality iDPC images can not be acquired. Besides, could the authors also provide a structure model of the triclinic P1 phase WO3 in Fig. 3?
8) What's the role of oxygen vacancy on the domain-wall conductivity? It will be great if the authors could provide insights into the electronic structure by performing electron energy loss spectroscopy (EELS) measurement (e.g. O K-edge, band gap etc.) at the domain wall.
9) The authors are advised to provide the image of fabricated Hall bar and the raw data of electrical measurements in supplementary file.
10) There are several errors or typos need to be corrected, such as those in the captions of Fig. 1 and Fig. S9.

Reviewer #3

(Remarks to the Author)
In this paper, the authors report a new polar triclinic phase in WO_3 thin films, which remains stable up to large thicknesses and elevated temperatures. The study is well-conceived and the experiments are thoroughly conducted, providing highly insightful findings. I believe this work merits publication in Nature Communications. However, several issues should be addressed prior to publication.
1. The authors emphasize the critical role of epitaxial shear strain in stabilizing the polar triclinic phase. To strengthen this claim, additional experimental and theoretical evidence should be provided. Furthermore, a structural diagram of the P1 phase, supported by theoretical calculations, would help confirm its proposed configuration.
2. The reviewer is wondering how the authors define the oxygen octahedral tilts in the WO3 film, especially the P1 phase.
3. In Fig. 1, PFM phase and amplitude images are shown for the sample rotated at 0° and 90°. The amplitude units in Figs. 1a and 1d are given in mV. To facilitate interpretation of the probe–sample interaction amplitude, it is recommended that the authors convert the amplitude scale to picometers (pm). For reference, please see the previous work by W.R. Geng et al. (Adv. Mater. 2022, 34, 2106396).
4. In the amplitude image of Fig. 1d, a noticeable contrast variation between the left and right sides appears to result from acquisition artifacts. It is recommended that the authors acquire a higher-quality replacement image. Furthermore, the phase and amplitude images in Figs. S3 and S4 exhibit skip/slip artifacts or are substantially influenced by surface topography, which should be addressed.
5. In Fig. 3, the ellipticity of oxygen columns is analyzed based on iDPC-STEM images. While the authors note that the ellipticity magnitude was measured through real-space image analysis, it would be helpful to provide a more detailed description of the image processing and data extraction procedures. Furthermore, the authors should include a discussion clarifying how the accuracy of the extracted ellipticity for the oxygen atomic columns was defined.
6. Figure 4 presents a detailed analysis of how the width of striped domains evolves with film thickness in WO_3 thin films-an intriguing finding. However, an important correlated phenomenon appears to have been overlooked. In Figs. 4a-c, as thickness increases, not only does the domain width change, but the in-plane polarization direction also rotates progressively. A careful analysis of the structural mechanism driving this rotation, along with a more detailed discussion, is needed. Additionally, it would be valuable to consider whether such in-plane polarization rotation could enable multi-state information storage applications.

Version 1:

Reviewer comments:

Reviewer #1

(Remarks to the Author)
I am pleased that the authors have provided additional explanations and analyses regarding the structure of triclinic WO3 deposited on YAlO3 (001), as well as the mechanism associated with epitaxial shear strain. The previously unclear aspects of polaronic conduction along the domain walls have also been satisfactorily clarified. Overall, the manuscript has been substantially improved, and I believe it is now suitable for publication in Nature Communications.

(Remarks on code availability)

Reviewer #2

(Remarks to the Author)
The authors have addressed most of my concerns and revised the manuscript accordingly. The revisions have enhanced its clarity, rigor and accessibility for a broad readership. I am pleased to recommend it for publication in Nature Communications.

(Remarks on code availability)

Reviewer #3

(Remarks to the Author)
The author has adequately addressed and revised most of the issues raised by the referee. However, given that the central innovation of this work is the discovery of a novel polar phase in epitaxial WO_3 thin films, it is essential to provide sufficient and convincing experimental and theoretical evidences to precisely determine the crystal structure of this phase in order to strengthen the scientific rigor and credibility of the study. Particularly, this work may serve as an important reference for future studies in this field, the referee strongly recommends that the authors provide more comprehensive experimental data or theoretical calculations to further validate the structure of the proposed new phase.
In addition, the precise determination of the new phase structure relies on high-quality experimental imaging data. Although the authors have presented iDPC-STEM images in Figure 3 to reveal the distribution of oxygen atoms in WO3, it should be noted that the iDPC-STEM imaging mode is highly sensitive to sample thickness. The visibility and contrast of the oxygen atomic columns can be significantly affected by thickness variations in the TEM specimen. Therefore, consistent with the suggestion of Referee 2, the referee recommends that the authors further optimize the TEM sample thickness in order to obtain higher-quality iDPC-STEM images. Such improvements would enable a more accurate visualization of the oxygen sublattice and provide clearer information on possible lattice distortions.
The referee recommends further revision and looks forward to reviewing a strengthened version of the manuscript.

(Remarks on code availability)

Open Access This Peer Review File is licensed under a Creative Commons Attribution 4.0 International License, which permits use, sharing, adaptation, distribution and reproduction in any medium or format, as long as you give appropriate credit to the original author(s) and the source, provide a link to the Creative Commons license, and indicate if changes were made.
In cases where reviewers are anonymous, credit should be given to 'Anonymous Referee' and the source.
The images or other third party material in this Peer Review File are included in the article's Creative Commons license, unless indicated otherwise in a credit line to the material. If material is not included in the article's Creative Commons license and your intended use is not permitted by statutory regulation or exceeds the permitted use, you will need to obtain permission directly from the copyright holder.
To view a copy of this license, visit https://creativecommons.org/licenses/by/4.0/

Response to reviewers

Polarity and anti-distortive polarons in WO_3 through epitaxial shear strain.

Ewout van der Veer, Martin F. Sarott*, Jack T. Eckstein, Stijn Feringa, Dennis van der Veen, Johanna van Gent González, Majid Ahmadi, Horatio R.J. Cox, Ellen M. Kiens, Gertjan Koster, Bart J. Kooi, Michael A. Carpenter, Ekhard K.H. Salje, and Beatriz Noheda*

(NCOMMS-25-93237)

We thank the reviewers for carefully reading our manuscript and for the encouraging comments as well as for the constructive questions. Below, our point-by-point response to the reviewers is shown in blue. We have addressed the comments of the reviewers and revised the manuscript, as well as the Supplementary Information, accordingly.

Reviewer #1 (Remarks to the Author):

Ewout van der Veer et al. report that a new polar phase of WO_3 is stabilized on a YAlO3(001) substrate through epitaxial shear strain, leading to polaronic conduction along domain walls where the distortive atomic mode is significantly suppressed. Strain engineering is a well-established strategy for tuning ferroic properties (magnetization and electrical polarization), electronic conduction, superconductivity, and for stabilizing metastable phases. While most prior studies have focused on longitudinal tensile or compressive strain originating from lattice mismatch, the authors emphasize the role of epitaxial shear strain in stabilizing a metastable polar phase. WO_3 is indeed a suitable platform for such investigation, as it exhibits diverse structural morphologies depending on temperature and substrate-induced strain. Moreover, phenomena such as bulk polarons, domain-wall conduction, and even domain-wall superconductivity have been reported in its low-temperature polar structures. By combining PFM, XRD, STEM, and transport measurements, the authors aim to demonstrate that epitaxial shear strain drives the emergence of a polar phase in WO_3.

The study is intriguing and potentially impactful. However, the manuscript requires clearer presentation of the experimental observations and stronger justification for the proposed shear-strain-induced phase stabilization and polaronic behavior. I outline several points below that I believe will help improve the clarity and rigor of the manuscript.

We appreciate the apt summary of our manuscript and gladly provide a response to the questions and comments below.

1) Clarification of PFM Phase Contrast (Figure 1b)

In Figure 1b, the phase difference between adjacent in-plane polarization domains appears to be approximately 40°, rather than the expected 180° for antiparallel polarization directions. Could the authors clarify why a 180° phase contrast is not observed in this measurement? Additional explanation regarding the PFM phase response in this system would be helpful.

Answer 1.1:

The non-180° lateral PFM phase contrast is the result of the background inherent to the experimental setup, a phenomenon that has been extensively studied by Soergel et al. (see https://doi.org/10.1063/1.2362984 or https://iopscience.iop.org/article/10.1088/0022-3727/44/46/464003). A common origin of this system-inherent background is a slight misalignment of the laser spot on the back of the cantilever, such that it is not perfectly located above the scanning-probe tip. In particular for weakly piezoelectric materials with a small piezoelectric amplitude, as is the case for our WO_3 films, this can lead to significant deviations from the ideal 180° phase contrast for oppositely polarized domains. This background can vary markedly between measurements and we have indeed measured a 180° lateral PFM phase contrast between the stripe domains on several occasions.

To clarify this point, we have added a note on page 5, lines 100-103 of the main manuscript and we now include additional lateral PFM data with 180° phase contrast in the Supplement in Fig. S3.

2) Interpretation of XRD Results

The XRD analysis is extensive, yet several aspects remain difficult to follow without additional explanation. Addressing the points below would significantly improve the clarity of the structural interpretation.

2-1) Diffraction Peak Separation in Figure 2b

In Figure 2b and line 146, the two diffraction peaks along the [100]_YAO direction maintain the same separation as the diffraction order increases. Could the authors clarify why these peaks do not exhibit the distinct triclinic domain behavior observed in Figure 1a?

Answer 1.2.1:

The reviewer correctly observed that the diffraction peaks viewed along the [010] axis of YAO, presented in Fig. 2a, show a four-fold triclinic splitting which increases with increasing diffraction order, a behavior which is not seen in the diffraction peaks viewed along the [100] axis.

To clarify why the magnitude of the splitting increases with increasing diffraction order along the [110] direction, but not the [1-10] direction, we have simulated the diffraction pattern of a triclinic unit cell with four domains as found in our films. A direct comparison of the sections of the reciprocal lattice shown in Fig. 2a,b with our simulation has been added as Fig. S9, showing increasing splitting of the four-fold reflections and not of the two-fold reflections.

We note that the primary evidence for a triclinic phase is not the presence of increasing peak splitting along only one direction (i.e. [001]), which could originate from two monoclinic domains, but rather splitting along *two* directions ([001] as well as 1-10]).

In the Supplementary Information in Fig. S7, we show a broader view of the reciprocal lattice of the sample. This can be compared with the simulations in Figure S8, where the simulation of both the four triclinic domains, as well as of an equivalent monoclinic unit cell with four domains, are included, further illustrating that a triclinic unit cell is required to explain the observed data.

We have clarified this point accordingly in the revised manuscript on page 9, line 155-169.

2-2) Methodology Behind Table 1 (Crystallographic Data)
Table 1 is central to distinguishing the proposed polar phase from previously reported WO_3 structures. However, the analytical procedure and statistical basis for extracting the crystallographic parameters are not sufficiently described. Providing details on the refinement method, fitting constraints, and error analysis would greatly strengthen the structural argument and clarify the triclinic peak splitting discussed in line 150.

Answer 1.2.2:
The unit cell parameters of the triclinic phase were obtained through peak finding, indexing and parameter refinements methods available in the Rigaku CrysAlisPro software (see Methods section), a commonly used software package in the single crystal x-ray diffraction community. The errors in the lattice parameters were obtained by calculating the standard deviation of the refined lattice parameters of the four indexed domains. The measured orientation matrices of the four domains and the twin relations between them have now been included in Supplementary Note S2.
It should be noted, however, that our claim of a triclinic phase does not stem from the measured unit cell parameters, but from the symmetry of the diffraction pattern (Figs. 2a/b, see Answer 1.2.1). Table 1 serves to demonstrate that the lattice parameters are different from those of the bulk monoclinic and triclinic phases, thereby providing additional evidence that our films have a distinct structure.

2-3) Explicit Assignment of Diffraction Peaks to Triclinic Domains
Although lines 164–169 and Figure 2d describe the triclinic domains, the interpretation remains difficult without explicit one-to-one assignments between diffraction peaks and real-space domain variants. While the splitting along q_x in Figure 2e can be attributed to the fact that gamma deviates from 90°, the manuscript does not explain the additional splitting along q_z. A more detailed description of the triclinic domain geometry, supported by Figure S7 and explicit peak-to-domain mapping, would greatly aid reader comprehension.

Answer 1.2.3:
Following the reviewer's request, we explicitly show which diffraction peaks belong to each of the four structural domains in the simulated diffraction patterns in Fig. S8 and S9. The relative orientations of the four structural domains in real-space are shown in Fig. S10.

The reviewer notes that the non-90° gamma angle is responsible for the diffraction peak splitting in the q_x direction. In the triclinic unit cell found in our films, the other two unit cell angles are also non-90°, which results in further splitting in the q_z direction. The combination of the three non-90° unit cell angles leads to four structural domains, each responsible for one of the four diffraction spots in figure 2a.

The diagram in Fig. 2d shows two of the domain variants, related to each other by a 180° rotation about the [100] axis of the YAO substrate. The other two domain variants are related to the ones in Fig. 2d by a 180° rotation about the [010] axis of YAO.

The four triclinic structural domains further correspond to the four distinct polar domain variants, as measured by PFM and illustrated in Fig. 1f.

We have added clarifying diagrams in the Supplementary Information in Fig. S8, S9, S10 and S12 and refer to them in the main text on page 9, lines 159, 168-169, 184-185.

2-4) Quantitative Description of Epitaxial Shear Strain

The non-orthogonal base angle of YAlO3 (91.5°) is presented as the origin of the epitaxial shear strain that stabilizes the polar phase (lines 122, 194). However, the discussion remains qualitative. A quantitative analysis of the shear strain imposed on WO_3—its magnitude, tensor components, and orientation—would significantly strengthen the argument and highlight the novelty of the shear-strain-driven mechanism.

Answer 1.2.4:

A quantitative description of the strain tensor in epitaxial films requires knowledge of the unstrained reference phase. In other words, in our case the question is which WO_3 phase gets epitaxially strained to give rise to the new triclinic phase. A common approach for the choice of the reference phase used, for instance, in Pertsev-type phase diagrams (see e.g. https://doi.org/10.1103/PhysRevB.67.054107), is to extrapolate the lattice parameters of a high-temperature, high-symmetry phase to room temperature.

Upon the suggestion of the reviewer, we have now calculated the in-plane linear and shear strains for two potential reference phases of WO_3: the bulk monoclinic phase that is stable at room temperature and a hypothetical cubic phase with the same unit-cell volume as the monoclinic structure. We then quantified the linear and shear strains between the WO_3 reference phases and the $YAlO_3$ substrate (see Marcus & Jona, https://doi.org/10.1103/PhysRevB.51.5263). This calculation yields linear strains of ϵ_{11} and ϵ_{22} along the [100] and [010] axes of the film, respectively, as well as the in-plane shear strain ϵ_{12}, listed in the table below. These calculations show that the magnitude of the shear strain is similar to that of the linear strains, suggesting that it plays a significant role in the epitaxial strain state of the film.

**YAO(001)**	Monoclinic reference cell	Cubic reference cell
ϵ_{11} (linear strain)	0.01229	-0.01494
ϵ_{22} (linear strain)	-0.01878	-0.01530
ϵ_{12} (shear strain)	0.01355	0.01318

A similar analysis is done of the strains imposed on the film when grown on the a different facet (the (110) facet) of the same substrate, where the triclinic phase is not observed (see Yun et al., https://doi.org/10.1038/s41467-020-18644-w). This analysis (see table below) shows zero shear strain, as the base angle between the in-plane pseudocubic axes is 90° for both the film and the substrate, unlike the (001) facet of $YAlO_3$ used in our work. We note that we calculated the strains separately for the A and B domains, as defined by Yun et al., as the epitaxial strain imposed on these domains are not equivalent by symmetry, which is the case for our films.

YAO(110)	Monoclinic reference cell A domain	Monoclinic reference cell B domain	Cubic reference cell
ϵ_{11} (linear strain)	-0.00490	0.01265	-0.01494
ϵ_{22} (linear strain)	-0.01878	-0.02629	-0.02248
ϵ_{12} (shear strain)	0	0	0

We now include these calculations with a supplementary note in the revised supplementary materials in section S1.

2-5) Temperature-Dependent Shear Strain and Structural Evolution
If temperature-dependent shear strain values for YAlO3(001) are available from literature or previous experiments, correlating them with the temperature-dependent structural evolution of WO_3 (Figure 2f) would deepen the discussion and reinforce the proposed mechanism.

Answer 1.2.5:
We have calculated the temperature-dependent lattice parameters of the bulk monoclinic phase of WO_3 and of $YAlO_3$ using coefficients of thermal expansion found in the literature (WO_3: https://doi.org/10.1107/S0365110X56001303, YAO: https://doi.org/10.1063/1.1944901). Using the same procedure outlined in answer 1.2.4, we then calculated the temperature dependence of the linear and shear epitaxial strains with respect to the bulk monoclinic reference structure, as well as the hypothetical cubic reference structure, which are shown in the figure below. In both cases, the variation of the different epitaxial strains with temperature is much smaller than their magnitude, showing that this variation does not play a significant role in the structural changes of the WO_3 film with temperature.

We have included this figure and a description in the revised supplementary information as Supplementary Note S1.

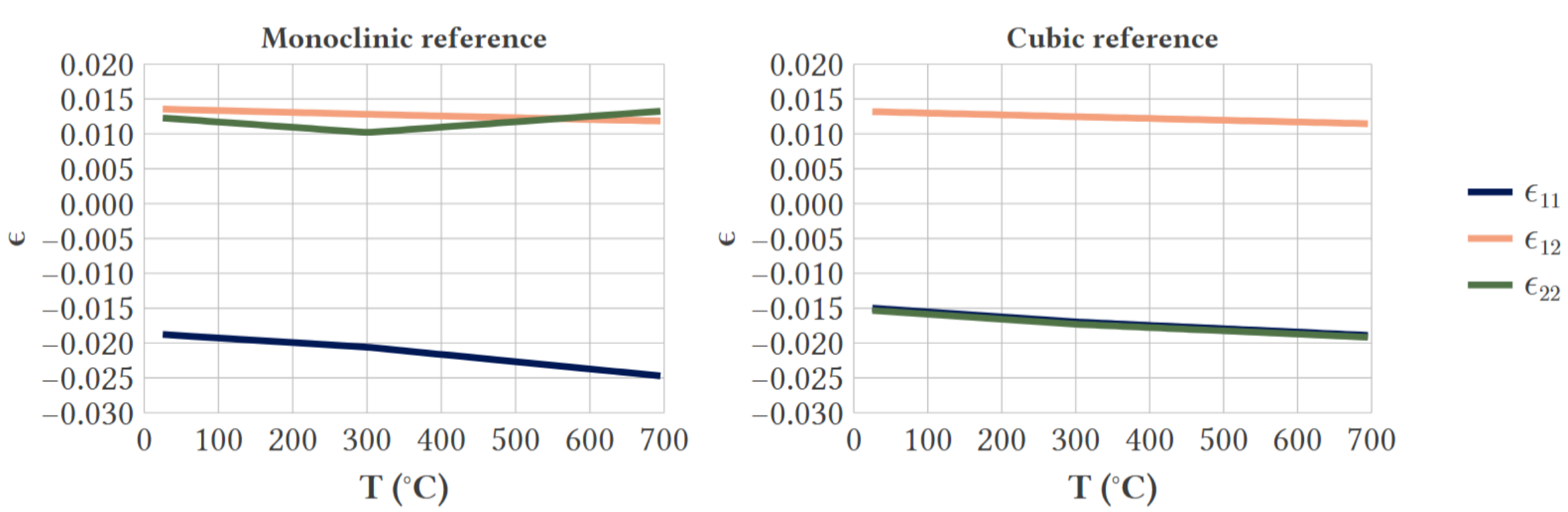

3) Directional Notations in Figure 4
In Figures 4a–c, adding directional labels would prevent potential misinterpretation that the polarization direction rotates with increasing film thickness. Clear axis or crystallographic direction annotations would improve readability.

Answer 1.3:
We thank the reviewer for the suggestion. In the revised version of Figs. 4a-c, we now include clear crystallographic axes with labels to improve readability.

4) Claim of "First Observation" of Anti-Distortive Polarons
The manuscript states that this work presents the first observation of an anti-distortive polaron (citing H. Hassani et al., Nat. Commun. 16, 1688 (2025)). However, among the four papers citing that work, an experimental study on WO_3 has already been published (G. Lee et al., Small 21, e04162 (2025)). The authors should clarify whether the present manuscript provides additional or more direct evidence that justifies emphasizing the "first" observation, or revise the claim accordingly.

Answer 1.4:
We believe our statement on the "first experimental observation" of anti-distortive-polaron-like transport to be justified for the following reasons.

Experimentally verifying anti-distortive polaron transport requires demonstrating two key points: 1) macroscopic electrical transport characteristics consistent with polaron transport (resistivity vs. temperature) and 2) a local coincidence of charge accumulation with the undoing of inherent structural distortions.

While in their paper Lee et al. analyze the electronic structure of cleaved WO_3 films using STM and ARPES, and find a polaronic two-dimensional electron gas at the film surface, they do not experimentally demonstrate the second key point, i.e. the undoing of inherent structural distortions. Only through DFT calculations, they show a reduction of bc-plane octahedral tilting at the surface.

In contrast, we show directly in real space, through STEM measurements, a clear reduction of all inherent structural distortions (both oxygen octahedral tilts and tungsten modulations), which spatially coincides with charge accumulation in cAFM. We then also show that this enhanced conductivity at the nanoscale manifests as macroscopic polaronic transport in Hall measurements.

We now refer to the paper by Lee on page 4, line 86 as reference 36.

5) Origin of Domain-Wall Conduction and the Definition of Anti-Distortive Polarons
The manuscript attributes the enhanced domain-wall conduction to anti-distortive polarons. However, the STEM images show that the domain-wall region appears undistorted even without charge transport, suggesting that the structure may already adopt an anti-distortive configuration. In this case, can the phenomenon still be classified as a polaron, which conventionally involves lattice distortion induced by charge localization? Alternatively, could the enhanced conduction be explained by the higher structural symmetry and reduced band gap at the domain wall, as suggested by structure-dependent calculations in Hassani et al.? Clarifying this conceptual distinction would strengthen the interpretation.

Answer 1.5:

The reviewer brings up an important point regarding the origin of the enhanced domain-wall conductivity. With our STEM analysis, we indeed do not directly image the atomic structure of a mobile polaron, which would be impossible with any state-of-the-art characterization technique, but rather the stable anti-distortive structure of the domain wall. Therefore, what we demonstrate here is a steady-state manifestation of anti-distortive charge trapping.

In a mobile anti-distortive polaron, the electronic energy lowering must overcompensate the anti-distortive and localisation energy costs so that it can appear spontaneously in bulk. At a domain wall, the undoing of the distortions is built into the structure, creating a local quantum well which can naturally trap free charges.

We further note that the bandgap lowering that results from the higher structural symmetry in the anti-distortive polaron, as shown by Hassani et al., is precisely the driving force behind the charge trapping. The concept of anti-distortive polarons is inherently linked to the evolution of the bandgap from phase to phase, as the authors of the Hassani paper explicitly point out in their conclusion.

To clarify the distinction between mobile anti-distortive polarons and anti-distortive charge trapping, we have expanded the discussion in the revised manuscript on page 17, lines 313-317.

5-1) Transport Perpendicular to Domain Walls (Figure 5c)

If conduction is dominated by polarons localized along domain walls, it is unclear how this mechanism would contribute to transport measured perpendicular to the stripe walls. In such a configuration, bulk conduction with higher activation energy would be expected to dominate. The authors should clarify whether polaronic transport is indeed relevant in this measurement geometry.

Answer 1.5.1:

Our macroscopic electrical transport characterization in Fig. 5c reveals that polaron transport dominates both along and perpendicular to the domain walls. As the referee correctly assumes and can be directly extracted from fitting the temperature-dependent resistivity data to a polaron transport model, in the configuration perpendicular to the domain walls the activation energy is indeed higher (0.141 eV) than parallel to the domain walls (0.106 eV). Therefore, the anti-distortive charge trapping at the domain walls gives rise to a reduced activation energy along the domain walls, but does not contribute to the polaronic conduction perpendicular to the domain walls.

To clarify this, we have edited the main manuscript on page 17, lines 322-326, and included the activation energies for polaron conduction as an inset of Fig. 5c.

Overall Assessment

The manuscript presents potentially significant findings regarding shear-strain-induced polar phases and domain-wall conduction in WO_3. Addressing the points above—particularly the structural analysis, quantitative strain discussion, and clarification of the polaronic mechanism—would substantially strengthen the manuscript and improve its clarity for readers.

Reviewer #2 (Remarks to the Author):

In the manuscript entitled "Polarity and anti-distortive polarons in WO_3 through epitaxial shear strain", van der Veer et al. reported the stabilization of a new polar phase in epitaxial WO_3 thin films grown on (001)-oriented YAO substrates. The authors determined the structure of this new phase at various scale by a combination of STEM, PFM and XRD. They also revealed via cAFM an enhanced conductivity at the stripe-domain walls featuring a pronounced reduction of the structural distortions. The conduction mechanism was pointed to anti-distortive polarons recently predicted in WO_3, further supported by the electrical transport measurements. Overall, this work is well designed and presented. I recommend this manuscript for publication in Nature Communications, given the following questions and suggestions are addressed.

We appreciate the reviewer's positive assessment of the quality, rigor, and significance of our work. Below our point-by-point response to the questions and suggestions are given.

1) A new polar and piezoelectric phase is stabilized in epitaxial WO_3 thin films. Have the authors performed secondary harmonic generation (SHG) measurements on the strained WO_3 thin films?

Answer 2.1:

We have performed optical SHG measurements in a 45° reflection geometry for two different probing wavelengths of the incident fundamental light (1200 nm or 1000 nm) and three different azimuthal sample orientations (0°, 45°, 90°). Furthermore, we have performed so-called polarimetry scans in which we systematically varied both the linear light polarization or the incident fundamental the and detected SHG light.

For all these measurement configurations, no SHG emission could be detected. Crucially, however, the absence of SHG emission does not preclude breaking of inversion symmetry. Integrated SHG measurements probe the average symmetry of an area defined by the spot size of the incident beam. In our case, we used a laser spot with a diameter of 250 µm. If the net polarization and, thus, the net inversion symmetry breaking within this probed area is zero, there will be no SHG emission. This is due to the fact that domains with an opposite orientation of the polarization emit SHG waves with a phase shift of 180°, which results in destructive interference. This is typically observed in compensated ferroelectrics, where the formation of oppositely polarized domains cancels the macroscopically-averaged net polarization in the system and, consequently, quenches the SHG emission (see for instance Figs. 1a/3 in https://doi.org/10.1038/s41467-022-30823-5 or Figs. 1c/4c in https://doi.org/10.1103/PhysRevLett.123.147601).

In our WO_3 films, as evident from the PFM data in Fig. 1, the population of the in-plane polarized domains is equal, which overall compensates any net in-plane polarization. Therefore, it is not surprising and, in fact, expected that we do not observe SHG emission in our films due to destructive interference.

In the revised version of the manuscript, we have added a statement on this on page 5, lines 117-120.

2) What's the relationship between Fig. 1(b) and Fig. S2(b)? Why are the two images presented in a mirror symmetric manner?

Answer 2.2:
We apologize for the oversight. The two images are indeed identical and only appear mirrored due to a difference in the plotting procedure. In the revised manuscript, we have now corrected this.

3) The statements in line 105-106: "the stripe domains reappear in the vertical channel" and line 110-111: "No domain contrast is found in vertical PFM (see Fig. S5)" are confusing, as the experiment details are not clear, such as fast scanning axis and sample orientation. For a broad readership, the authors are advised to schematically illustrate the line 104-106 and the inset in Fig. 1(d) in the manuscript or supplementary file, and to provide more experimental information of Fig. S5.

Answer 2.3:
The statement on line 105-106 refers to the appearance of the in-plane polarization in the vertical PFM signal due to buckling of the cantilever when it is oriented with its long axis parallel to the polarization direction.

The statements on line 110-111 refers to the vertical signal when the cantilever is oriented perpendicular to the polarization direction and, therefore, does not cause a buckling contrast.
To clarify, we have now added the cantilever orientation, crystallographic axes, and fast scanning axis via a double-headed arrow in the revised manuscript in the inset of Fig. 4d.

4) Could the authors perform the domain switching utilizing the mechanical trailing field of the PFM scanning tip?

Answer 2.4:
We have indeed performed attempts at manipulating the domain configuration using tip-induced pressure, however, without success. As part of future work, we are currently exploring the possibility to manipulate these polar domains through other electrical, mechanical, thermal, or optical means.

5) Regarding the macroscopic structural characterization, could the authors clarify "in one of four directions" in line 166-167? The statement in line 174-175 contradicts Fig. S8, as the bottom diffraction spot exists when temperature is above 600 ◦C. Do the bottom two spots disappear or merge in the first step phase transition around 600 ◦C (line 183)? A more clear and consistent description is necessary.

Answer 2.5:
We thank the reviewer for pointing out this inconsistency.
Regarding our statement on lines 167-167 on the existence of four triclinic structural domains and the four-fold splitting of the diffraction pattern along [010] of YAO, please refer to our answer to reviewer 1 (1.2.1).
Regarding the phase transition investigated in Figs. 2e/f and S13, we believe it is best understood as a combination of the following effects:

1) The two top diffraction spots visible at low temperature (arising from two of the triclinic domains) come closer together and merge together (Fig. 2f, left panel), reflecting a transition to a multi-domain higher-symmetry (possibly monoclinic or tetragonal) intermediate phase.
2) The top diffraction spots lose intensity (Fig. 2f, middle panel), reflecting the gradual disappearance of this intermediate phase.

3) The bottom diffraction spots lose intensity and disappear in a different temperature range (Fig. 2f, right panel).
4) Two new diffraction spots, belonging to the high-temperature phase, appear.

The presence of temperature hysteresis in the order parameters (Fig. 2f) and phase coexistence of the low- and high-temperature phases near the phase transition suggest that the transition is discontinuous/first-order.

To further study the nature of the intermediate phase requires large-volume reciprocal space mapping at elevated temperatures using synchrotron x-ray diffraction, which may be part of a follow-up publication.

We have clarified our description of this process in the manuscript on lines 175-188 and included a video of the diffraction pattern changing with temperature in the supplementary material.

6) Could the authors present in the supplementary file the RSM data of WO_3 films with a thickness larger than 250 nm?

Answer 2.6:
Prompted by the reviewer's request, we revisited the structural data for the thickest WO_3 films and found that even for the highest film thickness of 392 nm, the triclinic phase remains stable. Thus, our previous statement on lines 263-264 "For thicknesses larger than 250 nm, the film relaxes to the bulk monoclinic phase" was incorrect. We apologize for the mistake and note that none of the data and interpretation is affected by this.

To correct this error, we have revised the statement on lines 287-288 of the revised manuscript and added the RSM of the thickest WO_3 (392 nm) to the Supplementary Information in Fig. S16, clearly showing the persistence of the triclinic phase.

7) The iDPC-STEM image quality in Fig. 3 is inadequate to give accurate information on the oxygen octahedral tilt pattern of the triclinic P1 phase WO_3, maybe due to a thick TEM sample, giving large error on the fitting of oxygen atoms and the calculation of their ellipticity. Taking iDPC-STEM on a thinner TEM sample will help, or the authors could use ADF or ABF to visualize the oxygen atoms, if high quality iDPC images can not be acquired. Besides, could the authors also provide a structure model of the triclinic P1 phase WO_3 in Fig. 3?

Answer 2.7:
The observation of domain walls in these samples is difficult due to the subtle nature of the structural modulation associated with them and their relatively low density in the samples. The iDPC-STEM image presented in Fig. 3 is, despite the shortcomings pointed out by the reviewer, the clearest example of such a domain wall we have been able to capture. We nevertheless believe that the column-to-column modulation in the oxygen atomic columns can only be explained by a change of the octahedral tilting pattern and cannot originate from a sample thickness artifact, as is reflected in the small error relative to the layer-to-layer ellipticity difference outside of the domain wall region.

In the revised manuscript in Figs. 3c/f, we have now added the error bars to the calculated oxygen column ellipticities. We have further included an additional iDPC-STEM image in the supplementary information (Fig. S20), not using drift correction, still showing the same ellipticity modulations that are similarly inconsistent with the known bulk phases.

We share the reviewer's wish for a full structural model of the new triclinic phase, but the exact determination of such a low symmetry crystal structure on the basis of STEM images alone is intractable. To further elucidate our film's structure requires synchrotron x-ray diffraction experiments, which we are currently planning.

8) What's the role of oxygen vacancy on the domain-wall conductivity? It will be great if the authors could provide insights into the electronic structure by performing electron energy loss spectroscopy (EELS) measurement (e.g. O K-edge, band gap etc.) at the domain wall.

Answer 2.8:
We have performed preliminary EELS measurements, but not yet been able to conduct the measurements across domain walls due to their low density in the sample combined with sample drift during long EELS mapping. Our X-ray photoelectron spectroscopy measurements (Fig. S19), however, do not show any off-stoichiometry in the sample. This finding, combined with the observation of a polaronic conduction mechanism (section 2.5), suggest that oxygen vacancies are likely not responsible for the observed domain wall conduction. Further (EELS) studies are required to definitively exclude the presence of oxygen vacancies in the domain wall region.

9) The authors are advised to provide the image of fabricated Hall bar and the raw data of electrical measurements in the supplementary file.

Answer 2.9:
As suggested, we now include optical microscopy images of the fabricated Hall bars in Fig. S17 of the Supplementary Information. All the raw data of the electrical measurements are accessible in DataverseNL at https://doi.org/10.34894/QDT8NH. Furthermore, all scripts for the processing of the electrical data, including explanations for the data processing steps and fitting procedures are provided in https://github.com/hc3413/Electronic_properties_of_thin_films and cited in the submitted manuscript as reference 63.

10) There are several errors or typos that need to be corrected, such as those in the captions of Fig. 1 and Fig. S9.

Answer 2.10:
We appreciate the reviewer making us aware of these mistakes. We have thoroughly proof-read the entire manuscript, SI, and figure captions and corrected all typos.

Reviewer #3 (Remarks to the Author):

In this paper, the authors report a new polar triclinic phase in WO_3 thin films, which remains stable up to large thicknesses and elevated temperatures. The study is well-conceived and the experiments are thoroughly conducted, providing highly insightful findings. I believe this work merits publication in Nature Communications. However, several issues should be addressed prior to publication.

We thank the reviewer for acknowledging the significance of our findings. Our response to the questions and comments is provided below.

1. The authors emphasize the critical role of epitaxial shear strain in stabilizing the polar triclinic phase. To strengthen this claim, additional experimental and theoretical evidence should be provided. Furthermore, a structural diagram of the P1 phase, supported by theoretical calculations, would help confirm its proposed configuration.

Answer 3.1:

Regarding the critical role of the epitaxial shear strain, we refer the reviewer to our answers to reviewer 1 (1.2.4 and 1.2.5).

We acknowledge that a definitive theoretical basis for our proposed triclinic phase is still lacking. Our collaborators have performed preliminary DFT calculations by fixing the lattice parameters to those of the triclinic unit cell using the atomic positions of the known monoclinic phase. These calculations have, however, not yet been able to reproduce the observed triclinic symmetry. We propose the following possible explanations:

1) The observed triclinic phase is metastable and, as a result, its formation is highly dependent on the exact conditions during thin-film growth. These effects are not taken into account in a DFT calculation.
2) The DFT calculations thus far have only considered single-phase structures. Including the presence of the four triclinic structural domains in DFT calculations is computationally expensive, yet it may be highly relevant for the stabilization of the triclinic P1 phase observed in our films.
3) Other effects, such as the exact nature of the bonding interactions at the substrate-film interface may play a role in addition to the epitaxial shear strain mechanism described in our manuscript. Interfacial bonding was not explicitly included in DFT calculations performed so far.
4) The proximity of other stable phases (such as the known bulk monoclinic and triclinic P-1 phases), combined with computational inadequacies and inaccuracies in the starting guess for the atomic positions may favor the stabilization of those phases *in silico*.

Accounting for each of these effects in theoretical computations represents a significant body of work and a methodological advancement in itself. For this reason, we believe that providing such theoretical evidence is beyond the scope of this report and is more appropriate for a follow-up publication.

Furthermore, the determination of the exact atomic configuration of the novel triclinic phase, also requested by the reviewer, will significantly facilitate the theoretical investigation of this phase. As noted in answer 2.7, this requires synchrotron X-ray diffraction experiments, which cannot be performed immediately, but which we are planning.

2. The reviewer is wondering how the authors define the oxygen octahedral tilts in the WO_3 film, especially the P1 phase.

Answer 3.2:

The patterns of octahedral tilts in perovskite materials were systematically classified by Glazer (https://doi.org/10.1107/S0567740872007976). Following the Glazer notation, the $P2_1/n$ phase which is stable at room temperature in bulk WO_3 has an octahedral tilting pattern of $a^-b^+c^-$ and the P-1 phase which is stable just below room temperature in bulk WO_3 has a tilting pattern of $a^-b^-c^-$. From our STEM investigation, we are unable to precisely assign an octahedral tilt pattern in the Glazer notation due to the two-dimensional nature of the measured STEM projections in which the full three-dimensional structural information is lost. Nevertheless, Fig. S14 clearly demonstrates that the tilting pattern seen in our STEM images do not fit with the aforementioned known bulk structures of WO_3. We are planning synchrotron x-ray diffraction experiments to fully elucidate the complete crystal structure of our thin films.

The octahedral tilting patterns of the known bulk crystal structures have been added to the caption of Fig. S14.

3. In Fig. 1, PFM phase and amplitude images are shown for the sample rotated at 0° and 90°. The amplitude units in Figs. 1a and 1d are given in mV. To facilitate interpretation of the probe–sample interaction amplitude, it is recommended that the authors convert the amplitude scale to picometers (pm). For reference, please see the previous work by W.R. Geng et al. (Adv. Mater. 2022, 34, 2106396).

Answer 3.3:

All PFM data presented in Fig. 1 are lateral PFM images, where the image contrast originates from cantilever torsion rather than a cantilever deflection and causes a horizontal movement of the reflected laser spot on the quadrant photodiode. Converting a lateral PFM amplitude scale from millivolts to picometers is, therefore, not physically meaningful, because it does not originate from a vertical cantilever deflection. This is, in fact, consistent with PFM literature (see for instance the SI in https://doi.org/10.1038/s41467-020-15501-8), including the paper from Geng et al., where only vertical PFM amplitudes are reported in either arbitrary units or picometers.

Note that in the main text and the SI, we consistently report vertical PFM amplitudes in picometers and lateral PFM amplitudes in millivolts.

4. In the amplitude image of Fig. 1d, a noticeable contrast variation between the left and right sides appears to result from acquisition artifacts. It is recommended that the authors acquire a higher-quality replacement image. Furthermore, the phase and amplitude images in Figs. S3 and S4 exhibit skip/slip artifacts or are substantially influenced by surface topography, which should be addressed.

Answer 3.4:

In the revised manuscript in Figs. 1d/e, we have now replaced the lateral PFM images with a higher quality scan that does not feature lateral contrast variations in the PFM amplitude.

The scans shown in Figs. S3 and S4 indeed feature minor topography-induced skip/slip artifacts that manifest in the vertical PFM phase and amplitude images. We note, however, that they do not affect the lateral PFM phase and amplitude signals in Figs. 3b/c and 4b/c, and therefore, do not influence the interpretation of the data. We have replaced the scans in Fig. 1 with scans that do not exhibit any topography-induced artefacts.

5. In Fig. 3, the ellipticity of oxygen columns is analyzed based on iDPC-STEM images. While the authors note that the ellipticity magnitude was measured through real-space image analysis, it would be helpful to provide a more detailed description of the image processing and data extraction procedures. Furthermore, the authors should include a discussion clarifying how the accuracy of the extracted ellipticity for the oxygen atomic columns was defined.

Answer 3.5:
We have included a more extensive description of the real-space STEM analysis procedure in the Supplementary information (section S1). To quantify the accuracy of the ellipticity values, we have included error bars in Figs. 3c,f representing the standard errors of the ellipticity values.

6. Figure 4 presents a detailed analysis of how the width of striped domains evolves with film thickness in WO_3 thin films-an intriguing finding. However, an important correlated phenomenon appears to have been overlooked. In Figs. 4a-c, as thickness increases, not only does the domain width change, but the in-plane polarization direction also rotates progressively. A careful analysis of the structural mechanism driving this rotation, along with a more detailed discussion, is needed. Additionally, it would be valuable to consider whether such in-plane polarization rotation could enable multi-state information storage applications.

Answer 3.6:
Please refer to answer 1.3.

We apologize for the confusion. The in-plane polarization direction does not rotate with film thickness, but the three PFM images have been acquired with different sample orientations during PFM scanning. The XRD rocking curves (Fig. 4d), in which all samples have the same in-plane orientation, confirms that the polarization direction remains unchanged with the domain satellites only appearing along the (1-10) direction of WO_3.

To avoid confusion, in the revised manuscript, we have now added the crystallographic axes as insets of Figs. 4a-c and updated the figure caption.

Second response to reviewers

Polarity and anti-distortive polarons in WO_3 through epitaxial shear strain.

Ewout van der Veer, Martin F. Sarott*, Jack T. Eckstein, Stijn Feringa, Dennis van der Veen, Johanna van Gent González, Majid Ahmadi, Horatio R.J. Cox, Ellen M. Kiens, Gertjan Koster, Bart J. Kooi, Michael A. Carpenter, Ekhard K.H. Salje, and Beatriz Noheda*

(NCOMMS-25-93237)

We thank the reviewers again for reading our revised manuscript. All reviewers are satisfied with our answers to the previous questions and comments raised. Below is our point-by-point response to the comments by the reviewers, shown in blue. We have addressed the comments of the reviewers and revised the manuscript, as well as the Supplementary Information, accordingly.

Reviewer #1 (Remarks to the Author):
I am pleased that the authors have provided additional explanations and analyses regarding the structure of triclinic WO3 deposited on YAlO3 (001), as well as the mechanism associated with epitaxial shear strain. The previously unclear aspects of polaronic conduction along the domain walls have also been satisfactorily clarified. Overall, the manuscript has been substantially improved, and I believe it is now suitable for publication in Nature Communications.

We appreciate the positive feedback from the reviewer and the recommendation for publication in Nature Communications.

Reviewer #2 (Remarks to the Author):
The authors have addressed most of my concerns and revised the manuscript accordingly. The revisions have enhanced its clarity, rigor and accessibility for a broad readership. I am pleased to recommend it for publication in Nature Communications.

We thank the reviewer for their positive comments and their recommendation for publication in Nature Communications.

Reviewer #3 (Remarks to the Author):
The author has adequately addressed and revised most of the issues raised by the referee. However, given that the central innovation of this work is the discovery of a novel polar phase in epitaxial WO_3 thin films, it is essential to provide sufficient and convincing experimental and theoretical evidences to precisely determine the crystal structure of this phase in order to strengthen the scientific rigor and credibility of the study. Particularly, this work may serve as an important reference for future studies in this field, the referee strongly recommends that the authors provide more comprehensive experimental data or theoretical calculations to further validate the structure of the proposed new phase.

We believe that the evidence currently presented in the manuscript, consisting of

- large-scale reciprocal space mapping by x-ray diffraction to determine the unit cell of the film,
- measurement of the shift and nature of the observed phase transition,
- the imaging of the polar domain structure by PFM, and

- extensive characterization of the oxygen octahedral tilting by (iDPC)-STEM

unequivocally demonstrate the presence of a polar triclinic phase, different from the known phases of WO_3. We further show the potential for functionalization of this phase through anisotropic electrical conductivity of its domain walls.
The level of structural detail in our manuscript is rarely seen in high-impact first reports of novel material phases reported for the first time. Further characterization of the polarization direction in the proposed triclinic phase is only possible through highly demanding and time consuming synchrotron x-ray diffraction experiments and DFT calculations which require the involvement of new authors and are hindered by the difficulties we outlined in answer 3.1 of our response letter to the first set of reviewer comments. To address this point of the reviewer, we have added a statement on page 8, line 289 suggesting DFT and synchrotron-based XRD studies to further detail the structure, which are beyond the scope of this work.

Like the reviewer, we hope that our work can serve as a foundation for further studies into WO_3 thin films, which have recently attracted increased attention by the oxide electronic and thin film communities. As such, it is imperative that our manuscript be accepted for publication as soon as possible.

We respectfully draw the reviewer's attention to the young researchers who have contributed to this work. A further delay in publication, or a longer list of authors, would meaningfully hinder their academic progress. We hope for the understanding of the referee in this matter.

In addition, the precise determination of the new phase structure relies on high-quality experimental imaging data. Although the authors have presented iDPC-STEM images in Figure 3 to reveal the distribution of oxygen atoms in WO3, it should be noted that the iDPC-STEM imaging mode is highly sensitive to sample thickness. The visibility and contrast of the oxygen atomic columns can be significantly affected by thickness variations in the TEM specimen. Therefore, consistent with the suggestion of Referee 2, the referee recommends that the authors further optimize the TEM sample thickness in order to obtain higher-quality iDPC-STEM images. Such improvements would enable a more accurate visualization of the oxygen sublattice and provide clearer information on possible lattice distortions.

We understand the referee's concerns about the iDPC-STEM image quality in Fig. 3. In response to a similar comment by referee 2 (answer 2.7 of our previous response letter), we replaced Figure S20 by a new improved version. It now includes additional data on a thinner lamella, showing the same features already seen in Fig. 3 and the previous version of Fig. S20, supporting our claim of a triclinic polar phase.

The referee recommends further revision and looks forward to reviewing a strengthened version of the manuscript.

We thank the reviewer again for their critical reading of the manuscript. In light of the considerations laid out above and earlier statements by the reviewer (*"The study is well-conceived and the experiments are thoroughly conducted, providing highly insightful findings. I believe this work merits publication in Nature Communications"*), we interpret the remaining concerns of the reviewer as an understandable wish to know as much as possible about the structure of the new triclinic phase we report, rather than as a barrier for publication in *Nature Communications*.
